# Cu‒S Covalent Bonds Enable the Anchoring of Single‐atom Cu on Layered MoS_2_ for Highly Selective and Active Photothermal Catalytic Conversion of CO_2_−H_2_O to Ethanol

**DOI:** 10.1002/advs.202504167

**Published:** 2025-06-20

**Authors:** Yingao Luo, Gaoli Chen, Zhongliao Wang, Sujuan Zhang, Xiuzhen Zheng, Sugang Meng, Shifu Chen

**Affiliations:** ^1^ Key Laboratory of Green and Precise Synthetic Chemistry and Applications Ministry of Education College of Chemistry and Materials Science Huaibei Normal University Huaibei Anhui 235000 P. R. China

**Keywords:** CO_2_ reduction, Cu single atom, high selectivity, photothermal catalysis

## Abstract

The catalytic conversion of CO_2_ into high‐value C_2+_ products offers a sustainable path toward carbon neutrality. However, traditional photocatalytic and thermal catalytic methods face challenges like low selectivity and yields. Herein, a novel Cu/MoS_2_ photothermal catalyst is synthesized via a two‐step hydrothermal method, anchoring single‐atom Cu on layered MoS_2_ for CO_2_ and H_2_O reduction into C_2_ products (ethanol, acetylene, and ethane). Under optimal conditions (250 °C, 903 mW·cm^−2^, 320–780 nm), the Cu_5%_–MoS_2_ catalyst achieves an ethanol yield of 3.1 mmol·g^−1^·h^−1^, 4.6 times higher than blank MoS_2_. Mechanistic studies reveal that Cu improves light absorption and enhances CO_2_ adsorption and *COOH accumulation at MoS_2_ edge S sites, as confirmed by density functional theory (DFT) calculations. Mo–Cu dual sites stabilize *CHO intermediates, boosting C_2_ product selectivity. The synergistic photothermal effect accelerates charge migration and surface reactions. This work provides cost‐effective insights into photothermal CO_2_ conversion for fuel production.

## Graphical abstract

Cu_5%_‐MoS_2_ with dual Cu and Mo active sites enables efficient photothermal synergistic catalysis of the CO_2_–H_2_O system for ethanol production. The catalyst achieves a high ethanol yield of 3.1 mmol·g^−1^·h^−1^ with 88.35% selectivity, showcasing exceptional performance and selectivity under mild conditions.

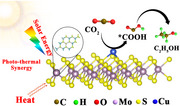

## 1 Introduction

The large‐scale emission of CO_2_ resulting from fossil fuel combustion is one of the primary contributors to global climate change.^[^ ^1^ ^]^ Accordingly, the development of efficient CO_2_ reduction technologies is a critical strategy for achieving renewable energy goals and carbon neutrality.^[^ ^2^ ^]^ Among these technologies, photothermal catalytic conversion of CO_2_ into value‐added fuels and chemicals shows considerable promise, offering broad prospects for practical application. In particular, ethanol has attracted significant attention in the field of CO_2_ reduction and conversion due to its high energy density (26.8 MJ·kg⁻¹) and advantages in transportation and storage.^[^ ^3^ ^]^

Photocatalysis offers several advantages, including mild reaction conditions, high selectivity, and environmental compatibility. However, its reaction rate is often constrained by the intensity and wavelength of the light source. In contrast, thermal catalysis exhibits superior reaction rates and conversion efficiencies. For instance, thermal energy can lower activation barriers or weaken the C═O bond, thereby accelerating reaction kinetics. Nevertheless, it typically requires elevated temperatures, which may induce undesirable side reactions and increase energy consumption. Photothermal catalysis, which integrates both photocatalytic and thermocatalytic processes, has emerged as a promising strategy to overcome these individual limitations. The synergistic interaction between light and heat not only enhances reaction rates and selectivity but also reduces the overall energy input. This dual‐modality approach facilitates catalytic activation at lower temperatures while leveraging light‐driven processes to improve both efficiency and environmental sustainability. As such, photothermal catalysis demonstrates great potential for practical applications. Despite these advantages, current photothermal catalytic technologies face significant challenges in the selective reduction of CO_2_ to high‐value multicarbon (C_2_⁺) products, such as ethanol.^[^ ^4^ ^]^ Consequently, ongoing research efforts are focused on the rational design of advanced catalysts and the development of effective strategies to steer product selectivity, with the ultimate goal of realizing efficient and controllable photothermal CO_2_‐to‐ethanol conversion.

Transition metal dichalcogenides (TMDs) have attracted significant research interest due to their low cost and high catalytic activity.^[^ ^5^ ^]^ Among them, molybdenum disulfide (MoS_2_) has been extensively employed in photocatalytic hydrogen evolution reactions (HER) owing to its unique optical and electronic properties.^[^ ^6^ ^]^ Studies have demonstrated that MoS_2_ serves as an efficient catalyst for water splitting during oxidation, wherein unsaturated sulfur atoms on its surface capture protons to form H_2_S.^[^ ^6^ ^]^ This process simultaneously exposes metal active sites with reducing properties, thereby accelerating the reaction rate. The most stable structure of monolayer MoS_2_ is a trigonal prismatic configuration, where the Mo layer is sandwiched between two sulfur layers in each S–Mo–S monolayer.^[^ ^7^ ^]^ Compared to conventional metallic catalysts, the d‐band center of Mo atoms exposed at the edges of MoS_2_ is closer to the Fermi level, indicating a strong interaction between the exposed Mo edge sites of MoS_2_ and adsorbates,^[^ ^8^ ^]^ which results in strong binding between MoS_2_ and key intermediates in CO_2_ reduction, such as CO* and COOH*.

However, the photocatalytic performance of pristine MoS_2_ is typically limited by its narrow light absorption range, low electrical conductivity, and rapid recombination of photogenerated charge carriers.^[^ ^9^ ^]^ Furthermore, the Mo edge active sites possess nearly identical coordination environments, which enhance dipole‒dipole interactions and disfavor the C–C coupling process.^[^ ^10^ ^]^ To overcome these limitations and improve the catalytic performance of MoS_2_ in CO_2_ reduction, constructing metal–semiconductor heterostructures has emerged as an effective strategy enhance the overall catalytic performance of semiconductors.^[^ ^11^ ^]^ Copper and its oxides (e.g., Cu, Cu_2_O, and CuO) are widely utilized in CO_2_ catalysis due to their loose d‐electrons, which facilitate multi‐electron transfer and promote C–C coupling reactions toward the formation of long‐chain hydrocarbons (C_2+_).^[^ ^12^ ^]^ MoS_2_ itself exhibits strong catalytic capabilities, particularly for deep CO_2_ reduction pathways.^[^ ^13^ ^]^ Unlike bulk MoS_2_, atomically dispersed Cu anchored at the sulfur edge sites of MoS_2_ enables direct bonding between oxygen atoms in CO_2_ and Cu atoms in Cu_5%_–MoS_2_, forming covalent interactions that effectively lower the activation energy barrier. The introduction of Cu not only modulates the electronic structure of MoS_2_ but also increases its specific surface area, thereby providing additional active sites and facilitating more efficient charge transfer pathways. This structural optimization drives the reaction preferentially toward the formation of liquid products. Owing to its inherent hydrogenation capability, MoS_2_ is particularly effective in further reducing CO_2_ intermediates to liquid products such as methanol and ethanol. The interfacial synergy between Cu and MoS_2_, enhances this hydrogenation process, thus boosting liquid product selectivity. In photothermal catalysis, single‐atom Cu catalysts offer significant advantages over their nanoparticle or cluster counterparts. Single‐atom Cu ensures 100% atomic utilization, with all active sites fully exposed and free from the agglomeration effects that commonly obscure catalytic surfaces. Moreover, single–atom Cu is chemically anchored to the support, offering enhanced thermal and structural stability even under harsh conditions such as elevated temperatures. This robustness enables single–atom Cu to more effectively harness photogenerated charge carriers in photothermal systems, thereby improving catalytic activity and product selectivity.

This study successfully prepared Cu/MoS_2_ composite materials via a facile hydrothermal method, achieving significant advancements in CO_2_ reduction through the incorporation of copper species and the implementation of a photothermal synergistic strategy. The work innovatively addresses three key theoretical challenges: i) it elucidated the modulation mechanism of copper doping on the electronic structure of MoS_2_, demonstrating that the Cu_5%_–MoS_2_ catalyst exhibits exceptional stability at 250 °C with substantially enhanced product yield compared to pure MoS_2_; ii) it clarified the mechanism by which copper species enhance visible light absorption and improve charge carrier separation efficiency; iii) it provided an in‐depth analysis of the photothermal synergistic catalytic mechanism, offering theoretical guidance for designing highly efficient non–noble metal–based CO_2_ reduction catalysts. This research not only developed a simple yet effective catalyst preparation strategy but also lays a solid foundation for the directional conversion of CO_2_ into high–value–added products.

## 2 Results and Discussion

### 2.1 Synthesis and Characterizations of Cu‐MoS_2_

**Figure 1:**
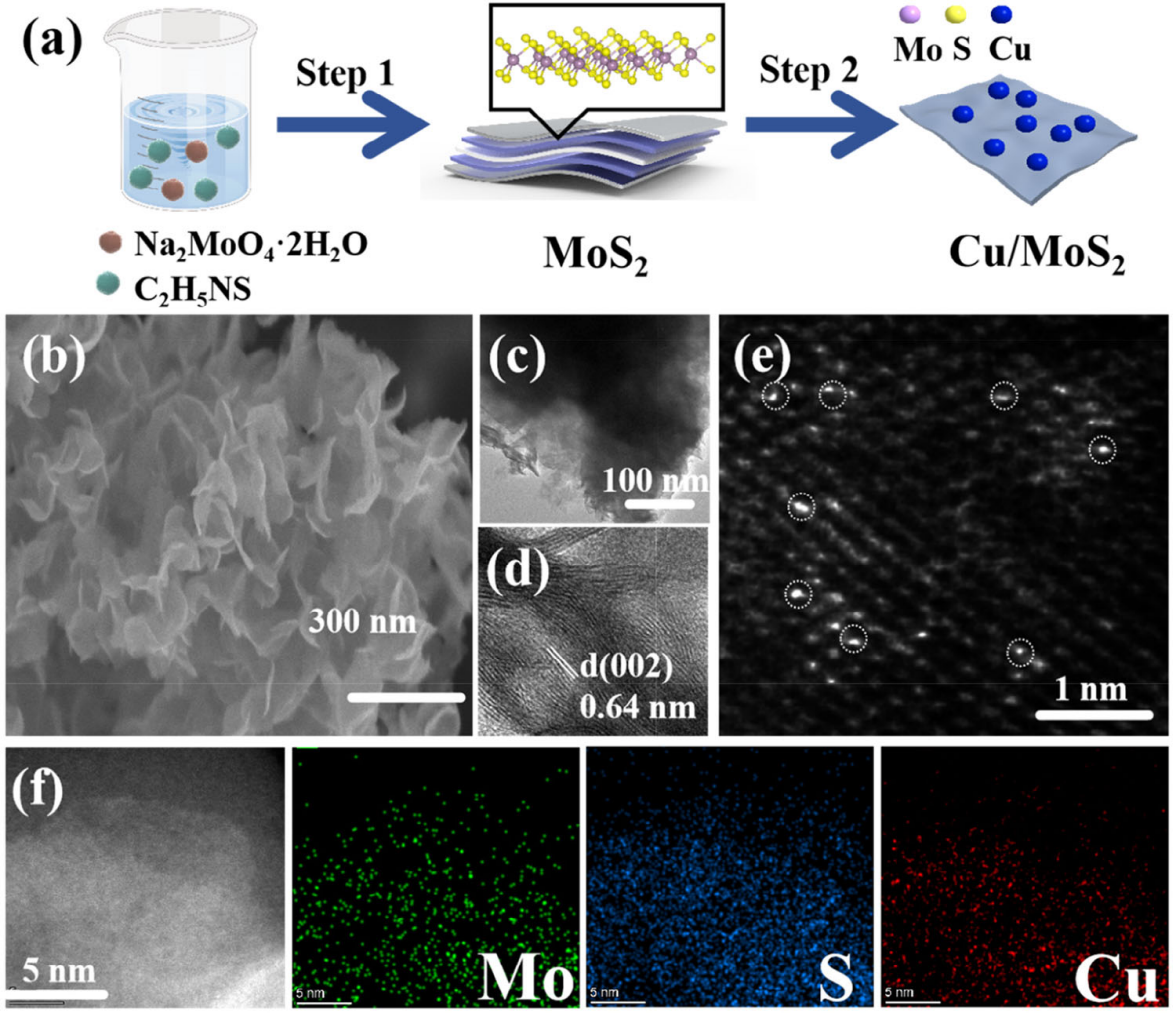
a) Schematic of Cu/MoS_2_ synthesis process, b–d) SEM, TEM, and HRTEM images of pure MoS_2_, e–h) SEM, TEM, HRTEM, and aberration‐corrected STEM images of Cu_5%_–MoS_2_, i) EDS elemental mapping of Cu_5%_–MoS_2_.

Based on the above theoretical design, Cu/ MoS_2_ was synthesized via a two‐step procedure, as illustrated in **Figure** **1**a. Detailed experimental conditions are provided in the Supporting Information (SI). The X‐ray diffraction (XRD) patterns of MoS₂ and Cu/MoS₂ are presented in Figure S1 (Supporting Information). The diffraction peaks at 14.22°, 33.57°, and 59.11° correspond to the (002), (100), and (110) planes of MoS_2_, respectively, in accordance with the standard JCPDS card No. 75–1539.^[^ ^14^ ^]^ Figure S2 (Supporting Information) presents the thermogravimetric (TG) curve of the Cu_5%_–MoS_2_ sample.^[^ ^15^ ^]^ The crystal structure and phase composition of pristine MoS_2_ and Cu_5%_–MoS_2_ were systematically analyzed. Figure 1 shows the morphology and microstructure of MoS_2_ and Cu‐loaded MoS_2_. The scanning electron microscope (SEM) and transmission electron microscope (TEM) images in Figure 1 show that Cu_5%_–MoS_2_ retains the sheet‐like structure of MoS_2_.^[^ ^16^ ^]^ Figure 1b displays the SEM image of pure MoS_2_ reveals that the material appears as nanosheets.

TEM analysis of the synthesized samples (Figure 1d,e) reveals that the nanosheets exhibit a certain degree of transparency, indicating their relatively thin structure. As shown in Figure 1f, the observed interlayer spacing of 0.62 nm corresponds to the (002) plane of MoS_2_. Notably, in Cu_5%_–MoS_2_, this spacing increases to ≈0.64 nm (Figure 1g), compared to that of pristine MoS_2_, suggesting lattice expansion upon Cu incorporation.^[^ ^17^ ^]^ Furthermore, the structural characteristics of MoS_2_ were investigated using Raman spectroscopy with a 532 nm laser (Figure S3, Supporting Information).^[^ ^18^ ^]^

To further investigate the specific distribution of copper atoms within MoS_2_ lattice, atomic‐resolution scanning transmission electron microscopy – annular dark field (STEM–ADF) imaging was performed (Figure 1h). No nanoparticles or clusters corresponding to metallic Cu or Cu‐based compounds were observed, confirming the presence of atomically dispersed copper species.^[^ ^19^ ^]^ This indicates that Cu is doped into MoS_2_ in the form of single atoms. Elemental mapping using energy dispersive spectrometer (EDS) confirmed the uniform distribution of Mo, S, and Cu in Cu_5%_–MoS_2_ (Figure 1i–m), and similarly, Mo and S were uniformly distributed in MoS_2_ (Figure S4, Supporting Information). Additionally, the X‐ray photoelectron spectroscopy (XPS) analysis detected Mo^4+^, S^2−^, and Cu⁺ species (Figures S5, Supporting Information).^[^ ^20^ ^]^ Additionally, N_2_ adsorption analysis (Figure S6, Supporting Information) showed that both MoS_2_ and Cu/MoS_2_ exhibit type І adsorption isotherms and H3‐type hysteresis loops.^[^ ^21^ ^]^

**Figure 2:**
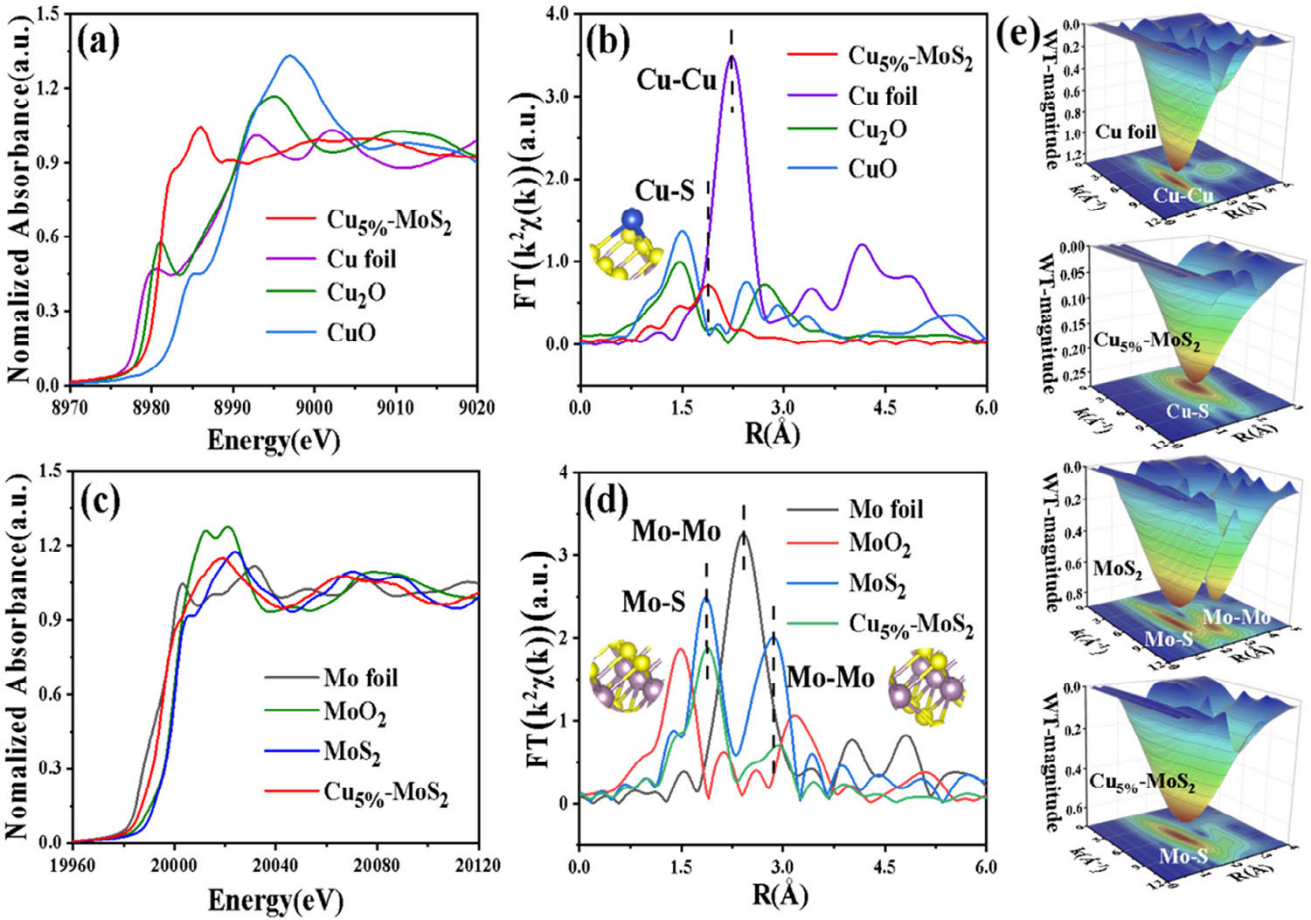
Cu K‐edge a) XANES and c) EXAFS analysis of Cu_5%_–MoS_2_, Mo K‐edge b) XANES and d) EXAFS analysis, e) full‐range EXAFS WT plots of Cu foil, MoS_2_, and Cu_5%_–MoS_2_.

The electronic structures of the Cu and Mo atoms were investigated via extended X‐ray absorption fine structure (EXAFS) and X‐ray absorption near‐edge structure (XANES) analyses. As shown in **Figure** **2**a, the Cu K‐edge XANES spectrum of Cu_5%_–MoS_2_ closely resembles that of Cu_2_O, suggesting that copper exists in a mixed oxidation state between +1 and +2. Moreover, a notable shift of the Mo K‐edge XANES spectrum of Cu_5%_–MoS_2_ was observed, confirming the uccessful incorporation of Cu into the MoS_2_ (Figure 2c). In the Fourier transform spectrum (Figure 2b), a strong peak at 1.88 Å is attributed primarily to Cu–S coordination. No correlation between the Cu–Cu coordination peaks was observed compared with those of copper foil, further confirming that the Cu atoms exist as single atoms in Cu_5%_–MoS_2_.^[^ ^22^ ^]^ Additionally, the EXAFS fitting results of the Cu foil and Mo foil are shown in Figures S8 and S9 (Supporting Information). The first coordination shell of the central Cu atom has a coordination number of 2, which corresponds to the coordination between Cu and the S atoms from the top and bottom layers of the MoS_2_ structure, with an average bond length of 2.31 Å. The fitting parameters for Cu are presented in **Table** **1** (the fitted parameters for Mo are presented in Table S1, Supporting Information). Furthermore, the intensity of the Mo─S and Mo─Mo bonds decreased compared with that of pure MoS_2_ (Figure 2d), which further verified the successful incorporation of Cu into MoS_2_. The K‐space EXAFS spectrum also confirmed the change in the coordination environment of Cu_5%_–MoS_2_ (Figures S10 and S11, Supporting Information). Using metal foils and metal oxides as standards (Figures S12–S15, Supporting Information), the wavelet change contour diagrams revealed different distributions of Cu in the Cu foil and Cu_5%_–MoS_2_, as well as distinct distributions of Mo in MoS_2_ and Cu_5%_–MoS_2_ (Figure 2e; Figures S16 and S17, Supporting Information).

**Table 1:** Cu K‐edge EXAFS curve fitting parameters obtained from Figure 3.

Sample	Shell	*CN* ^a)^	*R*[Å]^b)^	σ2 [Å^2^]^c)^	Δ*E*_0_[eV]^d)^	R factor
Cu	Cu‐S	2.40	2.31	0.011	1.56	0.004

^a)^ CN, coordination number;

^b)^ R, distance between absorber and backscatter atoms;

^c)^ σ^2^, Debye‐Waller factor to account for both thermal and structural disorders;

^d)^ ΔE_0_, inner potential correction; R factor indicates the goodness of the fit. Fitting range: 3.0<k(/Å)<12 and 1.0<R(Å)<3.

### 2.2 Study of Photothermal CO_2_ Conversion

**Figure 3:**
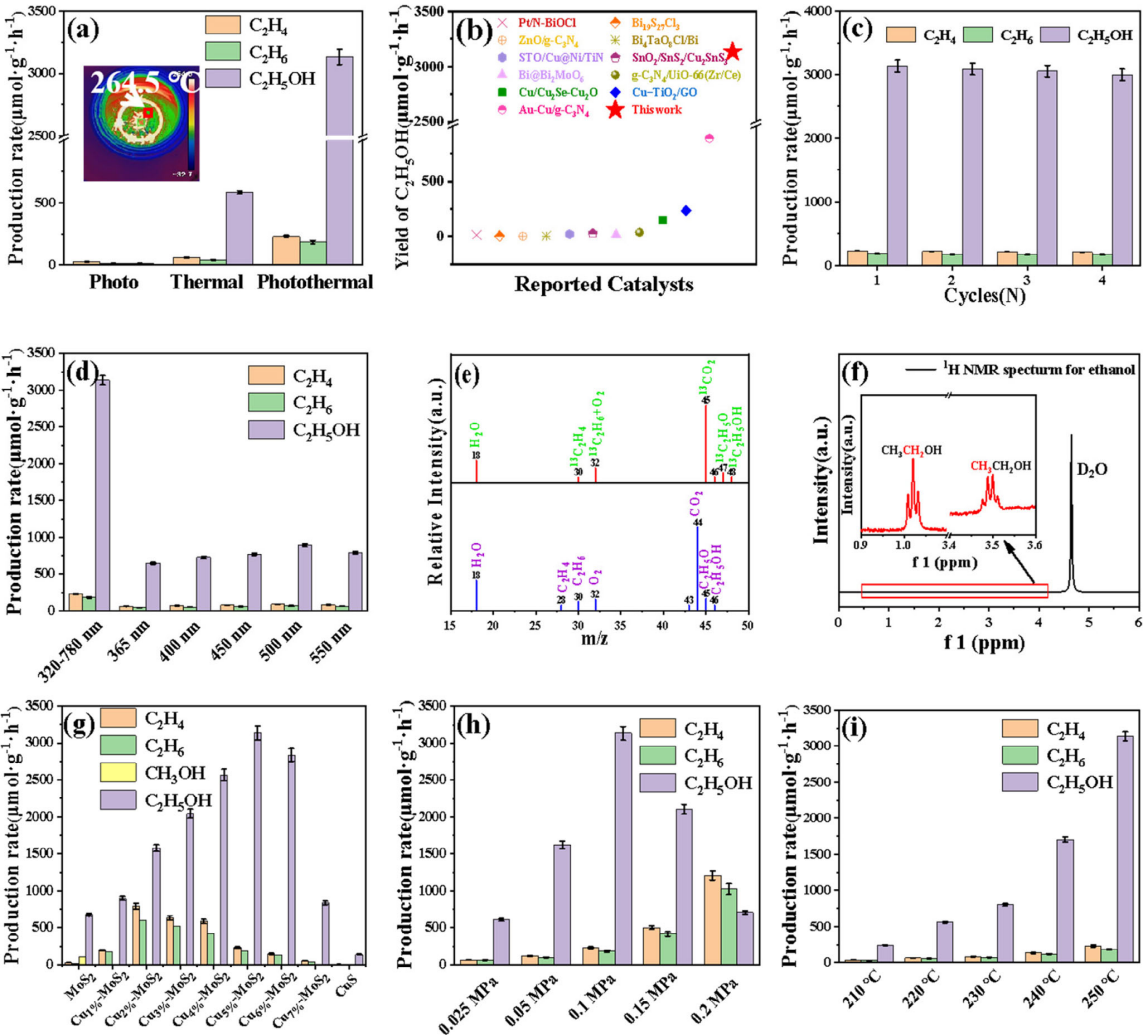
a) The yields of C_2_H_4_, C_2_H_6_, and C_2_H_5_OH from photothermal CO_2_ reduction under different catalysts and conditions, inset: thermal stability of Cu_5%_–MoS_2_ for 5 min under photothermal conditions; b) Comparison of ethanol performance in our work with that reported in previous literature, including both photocatalysis and photothermal catalysis;^[^ ^24^ ^]^ c) Cycling experiments; d) Effect of different wavelength light sources on catalytic activity; e) Mass spectrum of gaseous products after photothermal reduction of ^13^CO_2_ by Cu_5%_–MoS_2_ at 250 °C; f)) ^1^H NMR spectrum of ethanol produced after 10 h photothermal catalysis on Cu_5%_–MoS_2_. Catalytic performance of Cu/MoS_2_ with different Cu doping ratios under optimal conditions: g) Yield of C_2_H_4_, C_2_H_6_, CH_3_OH and C_2_H_5_OH. Catalytic activity of Cu_5%_–MoS_2_ at different temperatures:h) Yield of C_2_H_4_, C_2_H_6_ and C_2_H_5_OH. Catalytic activity of Cu_5%_–MoS_2_ under different pressures: i) Yield of C_2_H_4_, C_2_H_6_ and C_2_H_5_OH.

To further verify the photothermal catalytic activity of the Cu/MoS_2_ composite catalyst, a series of comparative experiments were conducted on the photothermal reduction of CO_2_ using MoS_2_ and MoS_2_ doped with different amounts of Cu. Catalytic measurements demonstrated that MoS_2_ has some activity in reducing CO_2_, yielding CH_3_OH and C_2_H_5_OH at rates of 107.72 and 676.12 µmol·g^−1^·h^−1^, respectively. However, Cu_5%_–MoS_2_ showed a significantly higher conversion rate of CO_2_, with the C_2_H_5_OH yield reaching 3.13 mmol·g^−1^·h^−1^ and no CH_3_OH production. The superior performance of Cu–MoS_2_ highlights its potential for photothermal catalysis in the production of C_2_H_5_OH from CO_2_. As shown in **Figure** **3**a, the Cu_5%_–MoS_2_ catalyst exhibits both photocatalytic and thermocatalytic activity and is capable of independently reducing CO_2_ under photothermal conditions. Notably, its photothermal catalytic performance significantly exceeds the simple arithmetic sum of its individual photocatalytic and thermocatalytic activities, highlighting a pronounced synergistic effect. This is because in the process of photothermal catalysis, the electrons in the catalyst are excited by light to form electron‒hole pairs. The electrons in this excited state can participate in the chemical reaction, and the light energy can provide additional energy for the CO_2_ reduction reaction, thereby reducing the activation energy of the reaction and increasing the reaction rate. The thermal energy can help overcome the activation energy of the reaction and promote the reaction. In addition, the rates of C_2_H_4_, C_2_H_6_, and C_2_H_5_OH production by photothermal catalysis were 230.16, 182.97, and 3134.6 µmol·g_cat_^−1^·h^−1^, respectively. Therefore, under the reaction conditions of 4 h light exposure, 250 °C, 0.1 MPa CO_2_, and 50 mg of catalyst, the selectivity of C_2_H_4_ was 6.49%, the selectivity of C_2_H_4_ was 5.16%, and the selectivity of C_2_H_5_OH was 88.35%. A comparison of ethanol production rates between this study and previous studies revealed that the ethanol production rate in this work surpassed those achieved by both photocatalytic and photothermal systems reported in the literature (Figure 3b).

Cycling tests are key indicators of catalyst stability.^[^ ^23^ ^]^ Cu_5%_–MoS_2_ was subjected to cycling tests, during which the catalyst was alternately washed multiple times with deionized water and ethanol after each reaction, thoroughly dried, and reused in subsequent reactions without adding fresh catalyst. The specific results are shown in Figure 3c. After four cycles of CO_2_ reduction under photothermal conditions, the catalytic performance of Cu_5%_–MoS_2_ did not significantly degrade, indicating good stability and sustained catalytic activity during photothermal CO_2_ reduction. The results from TEM, SEM, XRD, and EDS characterizations consistently indicate that there are no significant changes in the morphology or crystal structure of the Cu_5%_–MoS_2_ catalyst before and after the photothermal catalytic reaction (Figures S18 and S19, Supporting Information), thereby confirming its excellent structural stability. Simultaneously, the stability of individual MoS_2_ was investigated by calcining it at various temperatures under an argon atmosphere. The XRD patterns and activity test results (Figures S20 and S21, Supporting Information) demonstrate the excellent stability of the material.

To explore the effects of different light wavelengths on the catalytic performance, a series of experiments were conducted using bandpass filters of various wavelengths (Figure 3d, light intensities corresponding to different wavelengths are shown in Table S2, Supporting Information). The results revealed that Cu_5%_–MoS_2_ exhibited pronounced catalytic activity under irradiation at ≈500 nm. Catalytic reactions often require external energy input to overcome activation energy barriers. Photothermal catalysis synergistically combines photoexcitation and thermal effects, with light providing the photonic energy necessary for activation. Upon absorption, this energy generates electron–hole pairs in the catalyst, which subsequently drive the catalytic reaction. The range of photon energies that a catalyst material can absorb is dictated by its energy band structure. Typically, catalysts feature a conduction band (CB) and a valence band (VB). When the photon energy meets or exceeds the energy gap of the catalyst, it stimulates electrons in the valence band to transition to the conduction band, resulting in the formation of electron‒hole pairs. These electrons and holes play pivotal roles in photocatalytic reactions, suggesting that this particular wavelength (500 nm) acts as a threshold for the reaction wavelength involved in carbon dioxide reduction. To further reveal the origin of the product, we used isotopically labeled ^13^CO_2_ mass spectrometry, and when isotopically labeled ^13^CO_2_ was used as a reactant, only ^13^C_2_H_4_, ^13^C_2_H_6_ and ^13^C_2_H_5_OH were detected (Figure 3e), indicating that the product did indeed come from photothermal ^13^CO_2_ reduction. The presence of ethanol was confirmed by 1H NMR spectroscopy (Figure 3f).

The activity of Cu/MoS_2_ with varying Cu loadings was investigated, and the results indicated that the optimal composition is Cu_5%_–MoS_2_ (Figure 3g). The synergistic effect between Cu and MoS_2_ also played a significant role in enhancing the reaction selectivity. The introduction of copper not only improved electron transfer but also enhanced proton utilization, facilitating the hydrogenation reaction. In this context, the intermediate products generated from the reduction of CO_2_ with water are more likely to be deeply reduced into liquid products than simple gaseous products.

In the process of CO_2_ reduction, the duration of the catalytic reaction and the reaction temperature have a significant impact on catalytic performance. The results are shown in Figure 3h. Upon introducing CO_2_ into the system, the reaction pressure reached 0.1 MPa, under which the catalytic performance was optimized. As the pressure further increased, the solubility of CO_2_ in the reaction medium also rose, resulting in an increased number of CO_2_ molecules adsorbed and activated on the catalyst surface. This enhancement in adsorption and activation subsequently promoted more extensive CO_2_ reduction reactions. Furthermore, high pressure provides favorable conditions for deep reduction, as it enhances the adsorption of gaseous reactants (such as CO_2_ and H_2_O) and creates a better environment for proton transfer. Additionally, changes in the reaction energy barrier make deep reduction pathways (leading to liquid products) more favorable. As pressure increases, the change in free energy during the reaction tends to favor multi‐electron, proton‐coupled reduction reactions, producing liquid products such as ethanol. At lower pressures, the solubility of CO_2_ is reduced, resulting in shorter residence times for gas molecules on the catalyst surface, thus favoring the formation of gaseous products. The reaction pathway typically simplifies to a process involving fewer electron transfers. Under low‐pressure conditions, rapid adsorption and reduction rates result in predominantly gaseous products.

Through catalytic experiments conducted at different temperatures, the results shown in Figure 3i indicate that the photothermal catalytic effect is optimal at 250 °C. Notably, the catalytic activity is not significant below 230 °C, suggesting that this temperature may be the threshold for the reduction of CO_2_. Photothermal catalysis is a sophisticated process harnessing the synergistic power of light and thermal energy. Upon absorbing light energy, the catalyst's internal electrons undergo a transition to a higher energy state, known as the excited state, which typically necessitates a specific photon energy threshold. However, light energy alone is often insufficient to overcome the activation energy barriers required for chemical reactions, highlighting the critical role of thermal energy. Thermal energy acts as a complementary source of energy for light‐excited electrons. As the system temperature increases, the abundance of thermal energy increases, providing the impetus necessary for electrons to transition into the excited state. This enables the overcoming of high activation energies and fosters the generation of reactive species. Moreover, at relatively low temperatures, the electron‒hole pairs created by light excitation are prone to rapid recombination, a phenomenon that directly undermines the efficiency of the catalytic reaction. Conversely, a higher‐temperature environment can effectively increase the separation efficiency of electrons and holes, thereby extending their lifespan. This beneficial effect significantly amplifies the overall performance of the catalytic reaction. Hence, the brilliance of photothermal catalysis lies in its ingenious integration of light and thermal energy, optimizing the energy conversion pathway to achieve a marked increase in catalytic efficiency. At the same time, the influence of catalyst quality and reaction time on reaction activity was investigated (Figures S24 and S25, Supporting Information). The experimental results showed that 50 mg catalyst and 4 h catalyst were the best.

### 2.3 Mechanistic Insights into the CO_2_ Photothermal Reduction Processes

The separation and migration of photogenerated electron‒hole pairs can be evaluated via photoluminescence (PL) spectra and electrochemical measurements.^[^ ^25^ ^]^ Indicating a higher separation efficiency of photogenerated carriers in Cu/MoS_2_(Figures S26–S28, Supporting Information). We measured the electron paramagnetic resonance (EPR) spectra of MoS_2_ and Cu_5%_–MoS_2_ to study the ability of the catalysts to capture electrons (e⁻) and holes (h⁺). 2,2,6,6‐Tetramethylpiperidinooxy (TEMPO), a paramagnetic substance, exhibits a 1:1:1 triplet peak in EPR tests.^[^ ^26^ ^]^ Experimental results confirm that Cu_5%_–MoS_2_ generates more photogenerated e⁻ and h⁺ under light, enhancing the photocatalytic performance(Figure S29, Supporting Information).

**Figure 4:**
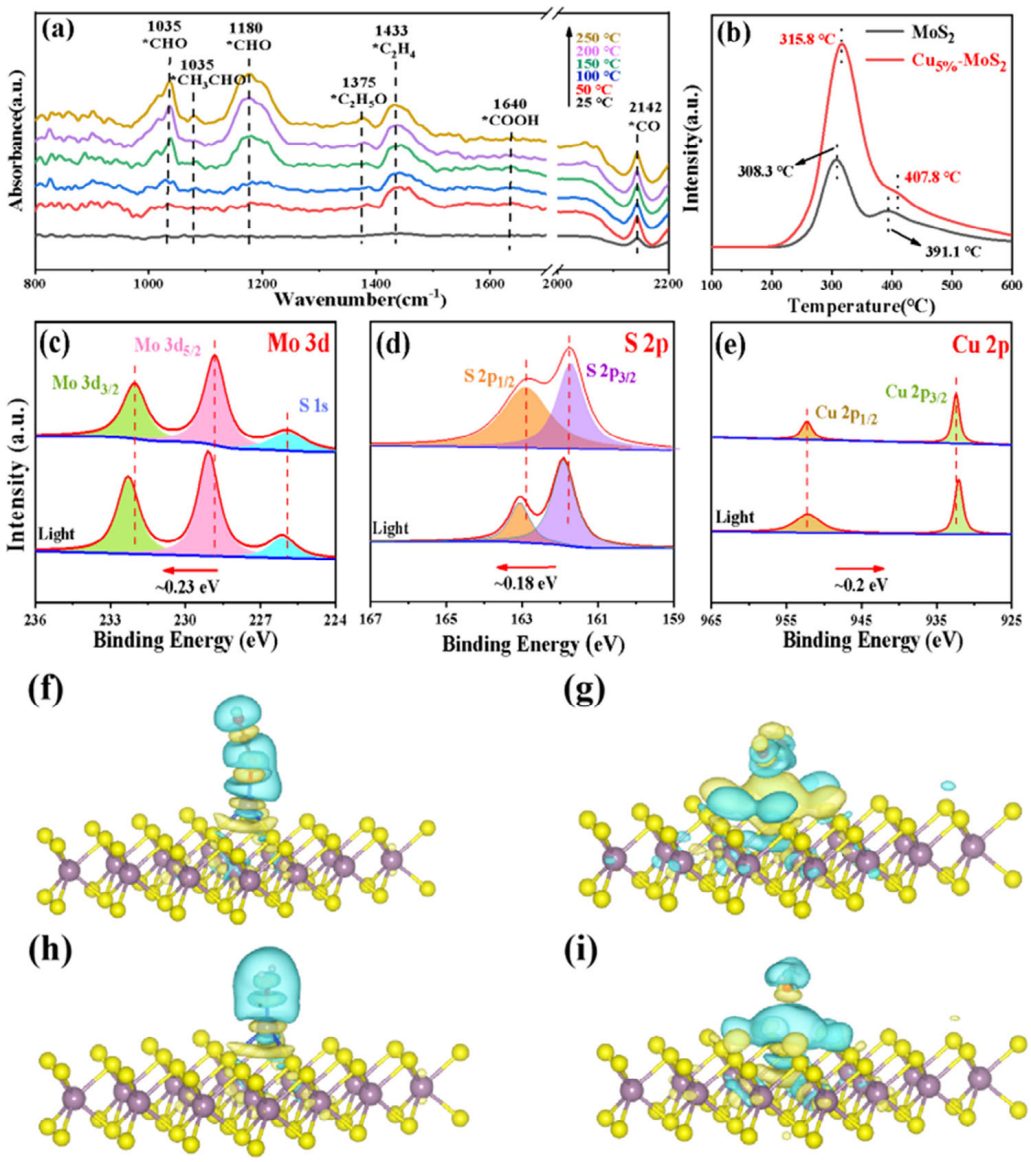
a) In situ FTIR spectra of co‐adsorption of CO_2_ and H_2_O on Cu_5%_–MoS_2_ under light irradiation; b) CO_2_–TPD results of MoS_2_ and Cu_5%_–MoS_2_. High‐resolution XPS analysis of c) Mo 3*d*, d) S 2*p*, and e) Cu 2*p*; f) Charge density difference of adsorbed CO_2_ molecules on Cu_5%_–MoS_2_ and g) MoS_2_ surfaces. h) Charge density difference of adsorbed H_2_O molecules on Cu_5%_–MoS_2_ and i) MoS_2_ surfaces. Purple, yellow, blue, red, and black spheres represent Mo, S, Cu, O, and C atoms, respectively.

To further understand the reaction mechanism, in situ fourier transform infrared spectroscopy (FTIR) spectroscopy was conducted during photothermal CO_2_ reduction for both MoS_2_ and Cu_5%_–MoS_2_ (**Figure** **4**a; Figure S30, Supporting Information). As shown in Figure 4a, an infrared peak at 1640 cm^−1^ was detected, attributed to the *COOH group, a key intermediate in CO_2_ reduction.^[^ ^27^ ^]^ The absorption peaks at 1180 and 1035 cm^−1^ correspond to *CHO and *CH_3_O groups, both of which are critical intermediates in the photoreduction of CO_2_ to hydrocarbons.^[^ ^28^ ^]^ The peaks at 2142 and 1035 cm^−1^ were assigned to adsorbed *CO and *CHO species, respectively. Additionally, a peak at 1375 cm^−1^ was attributed to adsorbed *CH_3_CH_2_O. Notably, the relative intensities of the *CO, *CHO, and *CH_3_CH_2_O peaks increased with temperature, indicating higher intermediate concentrations as the reaction progressed. The peak at 1180 cm^−1^ is assigned to the asymmetric stretching of *CH_2_O, the peak at 1443 cm^−1^ corresponds to the asymmetric stretching of *C_2_H_2_, and the peak at 1078 cm^−1^ corresponds to the characteristic stretching of *CH_3_CHO.

CO_2_ adsorption is crucial in CO_2_ conversion.^[^ ^29^ ^]^ Therefore, CO_2_ thermal programmed desorption (CO_2_–TPD) was used to investigate the CO_2_ adsorption capabilities of the catalysts. As shown in the CO_2_–TPD profile (Figure 4b), pure MoS_2_ exhibited two desorption peaks at 308.3 and 391.1 °C. After Cu loading, the desorption temperatures increased (315.8 and 407.8 °C), indicating increased CO_2_ adsorption.^[^ ^30^ ^]^ Additionally, the peak areas increased, reflecting increased adsorption capacity, suggesting that Cu doping provided more active sites.^[^ ^31^ ^]^ In Cu–doped MoS_2_ catalysts, Cu and Mo create dual active sites that possess distinctive electronic and geometric configurations. These dual active sites enable more efficient adsorption and activation of CO_2_ molecules. Notably, the incorporation of Cu may lead to certain regions on the MoS_2_ surface becoming more electron rich. This unequal distribution of electrons facilitates the adsorption of CO_2_ molecules and subsequent catalytic reactions.

XPS analysis was conducted to determine the surface chemical composition and electronic states of the samples. The results confirmed the presence of Mo, S, and Cu, which was consistent with the EDS analysis. Figure 4c–e displays the quasi‐in situ XPS spectra of Cu_5%_–MoS_2_. In Figure 4c, the XPS spectrum of Mo 3*d* shows S 1s (225.98 eV) and two Mo^4+^ peaks at Mo 3*d*_3/2_ (232.03 eV) and Mo 3*d*_5/2_ (228.85 eV), corresponding to the Mo^4+^ oxidation state. The binding energy of Mo increased by 0.23 eV after the reaction. Figure 4d shows the high–resolution XPS spectrum of S 2*p* in Cu_5%_–MoS_2_, with peaks at S 2*p*_1/2_ (162.86 eV) and S 2*p*_3/2_ (161.75 eV), where the S binding energy increased by 0.18 eV after the reaction. Figure 4e shows that the binding energy of Cu⁺^[^ ^32^ ^]^ decreases from Cu 2*p*_1/2_ (952.25 eV) and Cu 2*p*_3/2_ (932.39 eV) to Cu 2*p*_1/2_ (952.02 eV) and Cu 2*p*_3/2_ (932.18 eV) after the reaction. In summary, Mo and S increased in binding energy and lost electrons, whereas the binding energy of Cu decreased, indicating electron gain. Electrons were transferred from MoS_2_ to Cu.

The charge density difference (CDD) calculations revealed significant differences in the adsorption behavior of MoS_2_ and Cu_5%_–MoS_2_ (Figure 4f–g). The Bader charge of adsorbed *CO_2_ on MoS_2_ increased from 0.013 to 0.062 e on Cu_5%_‐MoS_2_, and the adsorption energy of Cu_5%_–MoS_2_ (−0.57 eV) was lower than that of MoS_2_ (−0.17 eV), which is consistent with the CO_2_–TPD results. The substantial charge accumulation around the CO_2_ molecules and the catalyst surface further confirmed the stronger CO_2_ affinity of Cu_5%_–MoS_2_. Similar adsorption and Bader charge analyses were conducted for H_2_O (Figure 4h–i). The Bader charge of H_2_O adsorbed on MoS_2_ increased from 0.016 to 0.058 e on Cu_5%_–MoS_2_, and the adsorption energy of Cu_5%_–MoS_2_ (−0.61 eV) was lower than that of MoS_2_ (−0.07 eV), suggesting that Cu doping facilitates H_2_O adsorption. Finally, we conducted a detailed study of the CO_2_ interfacial catalytic reduction process. DFT calculations have theoretically explored the adsorption and activation of CO_2_ reduction intermediates on Cu_5%_–MoS_2_ and MoS_2_ (Figures S30 and S31, Supporting Information), demonstrating that Cu_5%_–MoS_2_ has a stronger affinity for intermediates, promoting CO_2_ hydrogenation and C–C coupling reactions.

On the basis of the in situ FT–IR and DFT results, the CO_2_ reduction mechanism of Cu_5%_–MoS_2_ can be deduced as follows:

(1) $$CO_{2}\left(g\right)\to *CO_{2}$$

(2) $$*CO_{2}+H^{+}+e^{-}\to *\mathrm{COOH}$$

(3) $$*\mathrm{COOH}+H^{+}+e^{-}\to *\mathrm{CO}+H_{2}O$$

(4) $$*\mathrm{CO}+H^{+}+e^{-}\to *\mathrm{CHO}$$

(5) ∗CHO+∗CO→∗OCCOH

(6) $$*\mathrm{OCCOH}+H^{+}+e^{-}\to *\mathrm{CCO}+H_{2}O$$

(7) $$*\mathrm{CCO}+H^{+}+e^{-}\to *\mathrm{CHCO}$$

(8) $$*\mathrm{CHCO}+H^{+}+e^{-}\to *\mathrm{CHCHO}$$

(9) $$*\mathrm{CHCHO}+H^{+}+e^{-}\to *\mathrm{CCH}+H_{2}O$$

(10) $$*\mathrm{CCH}+H^{+}+e^{-}\to *\mathrm{CHCH}$$

(11) $$*\mathrm{CHCH}+H^{+}+e^{-}\to *CH_{2}\mathrm{CH}$$

(12) $$*CH_{2}\mathrm{CH}+H^{+}+e^{-}\to *C_{2}H_{4}↑$$

(13) $$*\mathrm{CHCO}+2H^{+}+2e^{-}\to *CH_{2}\mathrm{CHO}$$

(14) $$*CH_{2}\mathrm{CHO}+H^{+}+e^{-}\to *CH_{3}\mathrm{CHO}$$

(15) $$*CH_{3}\mathrm{CHO}+H^{+}+e^{-}\to *CH_{3}CH_{2}O$$

(16) $$*CH_{3}CH_{2}O+H^{+}+e^{-}\to *C_{2}H_{5}$$

(17) $$*C_{2}H_{5}+H^{+}+e^{-}\to C_{2}H_{6}↑+H_{2}O$$

(18) $$*CH_{3}CH_{2}O+H^{+}+e^{-}\to CH_{3}CH_{2}\mathrm{OH}$$

**Figure 5:**
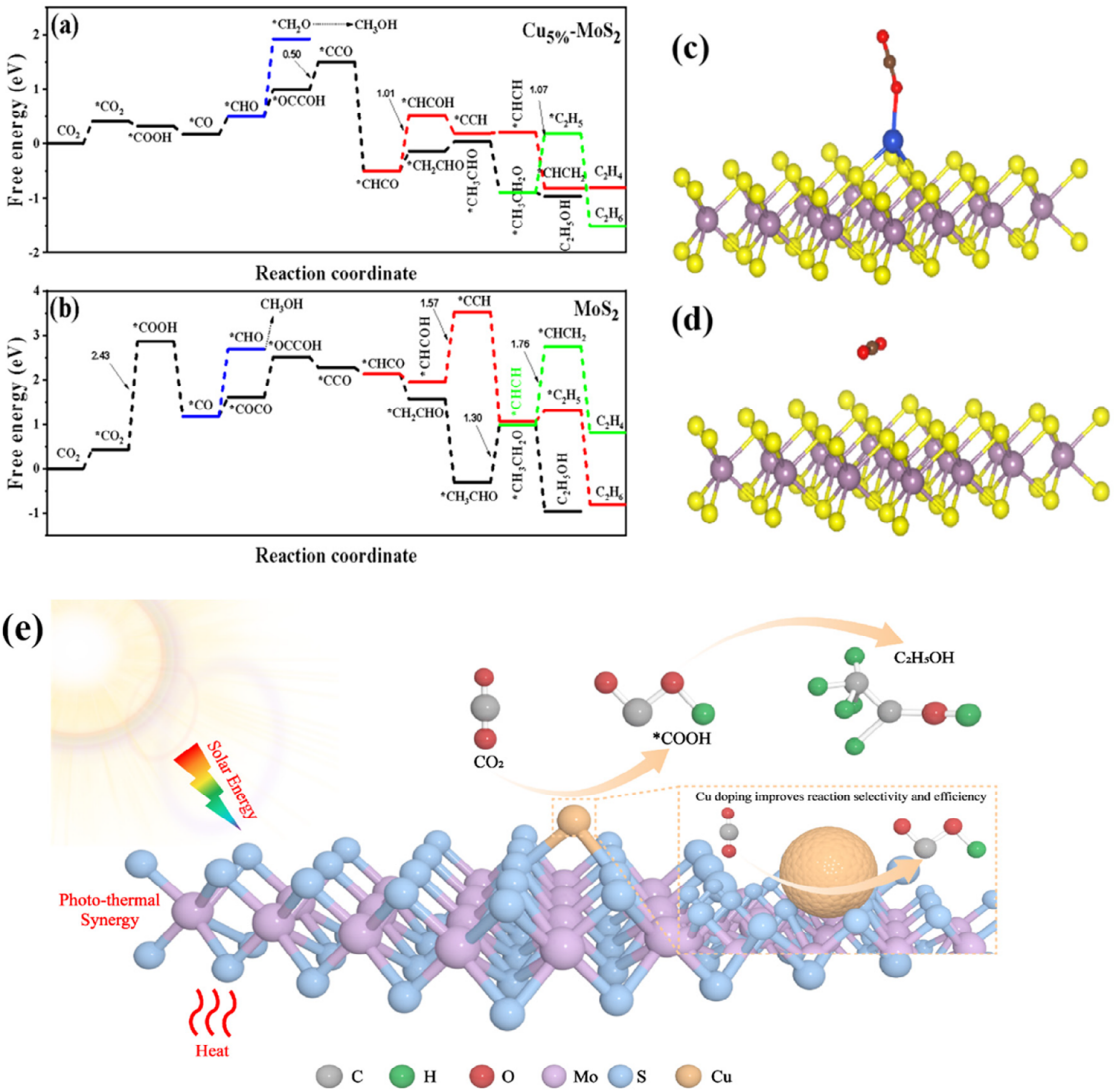
a) Gibbs free energy diagram for CO_2_ conversion on Cu_5%_–MoS_2_ and b) MoS_2_; c) Adsorption energy of CO_2_ molecules on Cu_5%_–MoS_2_ and d) MoS_2_. The purple, yellow, blue, red, and black spheres represent Mo, S, Cu, O, and C atoms, respectively; e) Schematic illustration of photothermal synergistic catalysis for CO_2_ reduction on Cu/MoS_2_.

On the basis of the reaction pathway derived from the in situ FT–IR results, the effects of Cu_5%_–MoS_2_ on the key steps of C–C coupling and desorption during the CO_2_ reduction reaction were studied through Gibbs free energy calculations (**Figure** **5**a–b). The results show that converting CO_2_ to *COOH on MoS_2_ requires overcoming an energy barrier of 2.84 eV, which is the rate‐limiting step. For Cu_5%_–MoS_2_, the introduction of Cu facilitates CO_2_ hydrogenation, lowering the energy barrier to a negative value. Additionally, the energy barrier for the hydrogenation of the *CO intermediate to form *CHO on Cu_5%_–MoS_2_ (+0.32 eV) is smaller than that for *CO on MoS_2_ (+1.51 eV). As a result, *CO tends to desorb more easily on Cu_5%_–MoS_2_. During the formation of *CCH from the *CHCO intermediate, Cu_5%_–MoS_2_ overcomes an energy barrier of 1.01 eV, which is much lower than the energy barrier of 1.57 eV for MoS_2_. Therefore, the formation of C_2_H_5_OH on the surface of Cu_5%_–MoS_2_ is kinetically more favorable than that of CH_3_OH. In summary, Cu_5%_–MoS_2_ improves the adsorption of reaction intermediates and reduces the energy barriers for CO_2_ reduction to C_2_H_5_OH.

We theoretically investigated the adsorption and activation behavior of CO_2_ on monolayer MoS_2_ and Cu_5%_–MoS_2_ surfaces via DFT calculations (Figure 5c,d). Unlike monolayer MoS_2_, the O atom of CO_2_ forms a covalent bond with the Cu atoms on the Cu_5%_–MoS_2_ surface. The bond length between the Cu and O atoms is 2.24 Å, whereas no covalent bonds form between the atoms in MoS_2_ and the C atoms. This leads to improved CO_2_ adsorption and activation.

Combining the experimental data with the theoretical calculations, the overall catalytic mechanism is illustrated in Figure 5e. Under the synergistic effect of light and heat, Cu enhances the affinity of MoS_2_ for CO_2_, lowering the CO_2_ adsorption energy and providing more active sites. Additionally, Cu‐loaded MoS_2_ effectively promotes charge separation, accelerates the C–C coupling process, and facilitates the accumulation of *COOH on the catalyst surface. This is a key step in ethanol formation.

## 3 Conclusion

This study establishes a dual‐function photocatalytic system for CO_2_ reduction coupled with H_2_O oxidation. Cu_5%_–MoS_2_, prepared via a hydrothermal method, has excellent photothermal synergy and outstanding ethanol production performance. Both the experimental and DFT results confirm that the introduction of Cu facilitates *CO coupling and promotes the conversion of *CH_3_CHO to *CH_3_CH_2_O. In contrast, pure MoS_2_ hinders C–C coupling and struggles to adsorb CO_2_ effectively. During the photothermal catalytic process, MoS_2_ absorbs light energy, generating photogenerated electrons and holes. These electrons and holes are separated and migrate to the surface of MoS_2_, aided by diffusion. Moreover, thermal energy enhances the adsorption and activation of reactant molecules on the MoS_2_ surface. When CO_2_ and H_2_O molecules are adsorbed on single Cu atoms, they react with the photogenerated electrons and holes, producing ethanol and other products. Our research offers valuable insights into the design of photothermal catalysts for converting CO_2_ into high–value C_2_ chemicals.

## 4 Experimental Section

### Synthesis of Cu/MoS_2_

Cu/MoS_2_ was synthesized via a two‐step procedure, as illustrated in Figure 1a. A one‐step hydrothermal method was used to prepare Cu/MoS_2_. Specifically, 0.25 g of sodium molybdate (Na_2_MoO_4_·2H_2_O) and 0.5 g of thioacetamide (C_2_H_5_NS) were dissolved in 40 mL of deionized water. After the mixture was stirred vigorously for 30 min, it was transferred to a stainless‐steel autoclave and reacted at 220 °C for 20 h. Upon completion, the precipitate was collected, washed, centrifuged, and dried to obtain pure MoS_2_. A certain amount of synthesized MoS_2_ (0.475 g) was then dispersed in 40 mL of deionized water, and 0.067 g of copper chloride (CuCl_2_·2H_2_O) was added. After 10 min of ultrasonication for uniform dispersion, the reaction proceeded at 220 °C for 6 h. The composite was then purified with deionized water to remove residual chemicals, and the black precipitate was dried under vacuum. The resulting sample was designated Cu/MoS_2_. ICP tests are shown in Table S3 (Supporting Information).

### Photothermal Catalytic Activity Testing

The photothermal catalytic activity was tested in a high‐temperature, high‐pressure visual reactor. First, 50 mg of the catalyst was uniformly dispersed in 10 mL of ultrapure water, and the reactor was sealed immediately. The gas inlet was then connected to a CO_2_ cylinder, and the gas outlet was opened to allow CO_2_ to purge for 15 min, ensuring the complete removal of other gases in the reactor. The outlet was then closed, and the internal pressure was precisely adjusted to 0.1 MPa. Next, the mixture was stirred in the dark for 30 min to ensure that adsorption‒desorption equilibrium was achieved under dark conditions. After the dark reaction, a xenon lamp was turned on to start illumination, and the temperature control program was initiated simultaneously. The reactor temperature was stabilized at 250 °C for 4 h. After the reaction, the gas outlet was connected directly to a gas chromatograph (Shimadzu GC–2014C) for precise quantitative analysis of the gaseous products. The mixture was centrifuged, and the supernatant was analyzed via gas chromatography (FuLi 979 011) for quantitative analysis of the liquid products.

### Computational Detail

DFT calculations were conducted through the Vienna ab initio Simulation Package (VASP) with the projector augment wave method.^[^ ^33^ ^]^ Generalized gradient approximation of the Perdew‐Burke‐Ernzerhof (PBE) functional was used as the exchange‐correlation functional.^[^ ^34^ ^]^ The Brillouin zone was sampled with 2 × 2 × 1 K points for surface calculation.^[^ ^35^ ^]^ The cutoff energy was set as 500 eV, and structure relaxation was performed until the convergence criteria of energy and force reached 1 × 10^−5^ eV and 0.02 eV Å^−1^, respectively. A vacuum layer of 15 Å was constructed to eliminate interactions between periodic structures of surface models. The van der Waals (vdW) interaction was amended by the zero damping DFT–D3 method of Grimme.^[^ ^36^ ^]^

The adsorption energy (ΔEads) of adsorbate adsorption on surface is defined as

(19) $$\Delta E_{ads}=E\left(*adsorbate\right)-E\left(*\right)-E\left(adsorbate\right)$$

where *E*(*adsorbate) and *E*(*) are the total energy of surface systems with and without adsorbate, respectively, *E*(adsorbate) is the energy of an isolated adsorbate. According to this definition, negative adsorption energy suggests that the adsorption process is exothermic and the adsorption system is thermodynamically stable. Contrarily, a positive value corresponds to an endothermic and unstable adsorption.

The Gibbs free energy was calculated as ΔG = Δ*E* + ΔEZPE −TΔS, where the ΔE, ΔEZPE, and ΔS are electronic energy, zero‐point energy, and entropy difference between products and reactants. The zero‐point energies of isolated and absorbed intermediate products were calculated from the frequency analysis.^[^ ^36^ ^]^ The vibrational frequencies and entropies of molecules in the gas phase were obtained from the National Institute of Standards and Technology (NIST) database.^[^ ^37^ ^]^

## Conflict of Interest

The authors declare no conflict of interest.

## Author Contributions

Y.L. wrote the original draft, contributed to formal analysis, and data curation. G.C. contributed to funding acquisition, data curation, visualization, wrote the original draft, review and performed editing. S.Z. performed funding acquisition, acquired resources, wrote the original draft, review and performed editing. Z.W. performed formal analysis and data curation. X.Z. developed software. S.M. performed funding acquisition. S.C. performed funding acquisition, supervision, wrote the review and performed editing.

## Supporting information



Supporting Information

## Data Availability

The data that support the findings of this study are available from the corresponding author upon reasonable request.
