# Supplementary material for: Cu‒S Covalent Bonds Enable the Anchoring of Single‐atom Cu on Layered MoS2 for Highly Selective and Active Photothermal Catalytic Conversion of CO2−H2O to Ethanol
Source: Adv Sci (Weinh). 2025 Jun 20;12(34):e04167. doi: 10.1002/advs.202504167 (PMC12442649; doi:10.1002/advs.202504167)
Supplement: Supplementary file 1 — Supporting Information [file ADVS-12-e04167-s001.docx]

Supporting Information

**Cu‒S covalent bonds enable** **the anchoring of** **single-atom Cu on layered MoS_2_ for highly selective and active photothermal catalytic conversion of CO****_2_−H_2_O to ethanol**

Yingao Luo, Gaoli Chen*, Zhongliao Wang, Sujuan Zhang*, Xiuzhen Zheng, Sugang Meng^a^, Shifu Chen*

**Materials**

The main reagents used for catalyst preparation and activity tests are as follows: sodium molybdate dihydrate (Na_2_MoO_4_·2H_2_O, 99% RG, Adamas); thioacetamide (C_2_H_5_NS, 99% RG, Adamas); copper(II) chloride dihydrate (CuCl_2_·2H_2_O, 99% RG, Adamas); ethanol (C_2_H_5_OH, 99.5% AR, Greagent); methanol (CH_3_OH, 99.5% AR, Greagent); 2,2,6,6-tetramethylpiperidin-1-oxyl (TEMPO, C_9_H_18_NO, 99.5% AR, Adamas); and acetonitrile (CH_3_CN, 99.5% AR, Greagent).

All the chemical reagents used in this study were of analytical grade and used without further purification, and deionized water was used in all the experiments.

**Experimental instruments**

Powder X-ray diffraction (XRD) patterns were recorded on a Bruker D8 Advance X-ray diffractometer with a scanning range of 10–70° and a scanning rate of 10°/min. Ultraviolet–visible diffuse reflectance spectroscopy (UV–vis DRS) was performed via a TU–1950 visible–near-infrared spectrophotometer (TU1950, Persee), with BaSO_4_ as the reference. Scanning electron microscopy (SEM) (FEI Quanta 650) was used to analyze the microstructure and surface characteristics of the samples. Transmission electron microscopy (TEM) and high-resolution transmission electron microscopy (HRTEM) imaging were performed on a JEM–2100 transmission electron microscope with an accelerating voltage of 200 kV. Synchrotron radiation was measured at Spring 8. The Brunauer–Emmett–Teller (BET) surface area of the samples was analyzed via N_2_ adsorption‒desorption on an ASAP 2460 system. X-ray photoelectron spectroscopy (XPS) measurements were carried out on an AXIS SUPRA+ photoelectron spectrometer. All binding energies were calibrated relative to the C 1 s peak of surface amorphous carbon at 284.6 eV. Electrochemical impedance spectroscopy (EIS) was used to measure the transfer resistance of the prepared MoS_2_ and Cu_5%_–MoS_2_ samples. The experiments were conducted on a CHI-660E electrochemical workstation (CH Instruments) using the prepared samples (5 mm × 5 mm, FTO/MoS_2_) as the working electrode, a Ag/AgCl electrode (in 3 M KCl solution) as the reference electrode, and a platinum wire as the counter electrode in a standard three-electrode cell. The thin-film working electrodes were prepared by drop-casting the sample/H_2_O suspension onto FTO glass substrates and drying overnight in air. The electrolyte used was an aqueous solution containing K_3_[Fe(CN)_6_] (0.01 M), K_4_[Fe(CN)_6_] (0.01 M), and 0.05 M KCl.

**
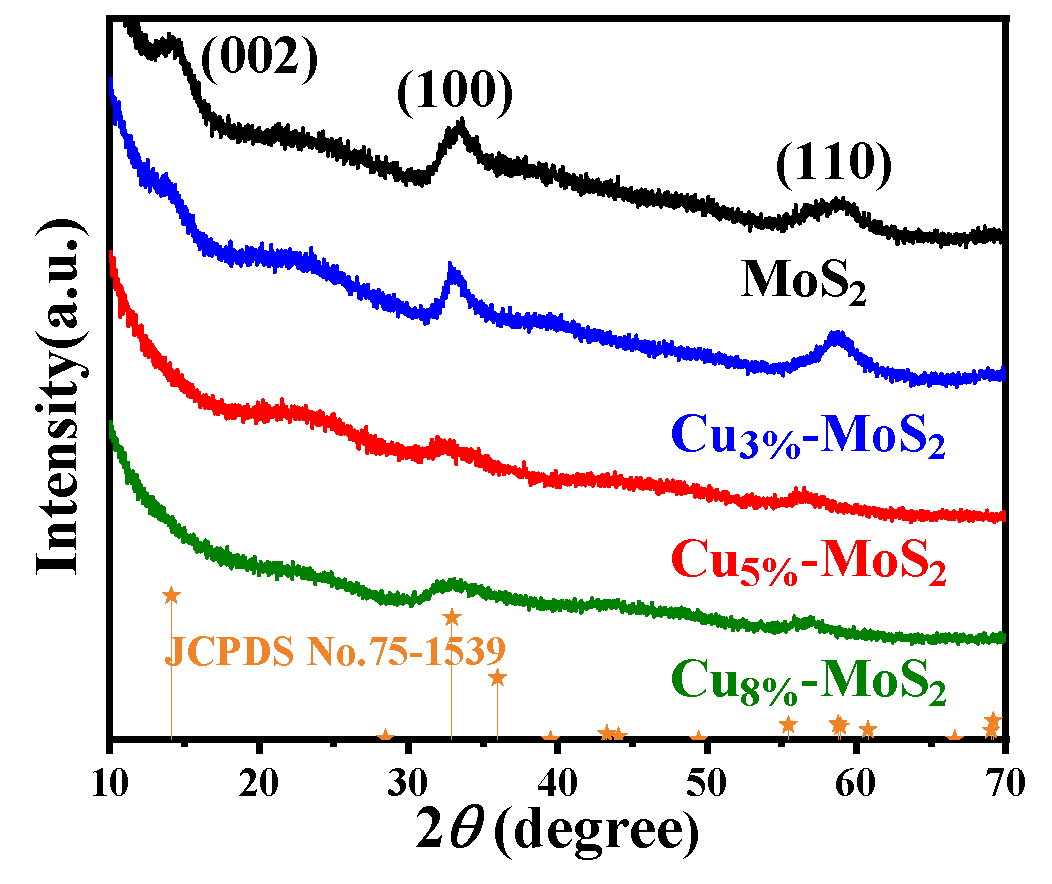
**

**Figure S1.** XRD patterns of different samples.

The XRD patterns of MoS_2_ and Cu/MoS_2_ are shown in Figure S1. The diffraction peaks at 14.22°, 33.57°, and 59.11° correspond to the (002), (100), and (110) planes of MoS_2_ (JCPDS No. 75–1539), indicating that the synthesized MoS_2_ is in the 2H phase. After Cu loading, as the Cu content increased, the peak intensity gradually decreased, and the peaks became broader, suggesting that Cu doping resulted in a reduction in the grain size.

**
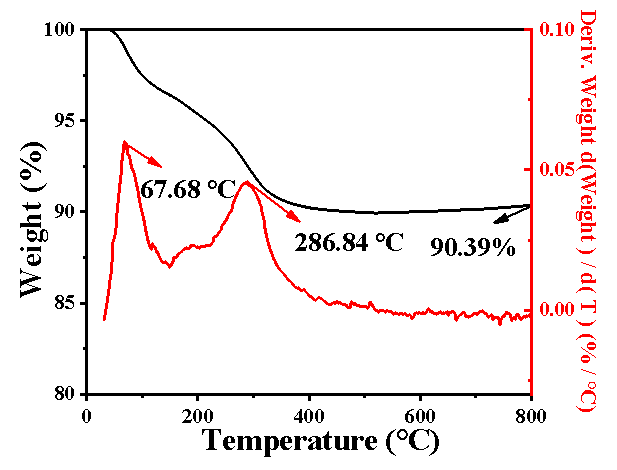
**

**Figure S2.** Thermogravimetric analysis curve of Cu_5%_–MoS_2_ in an Ar atmosphere.

Figure S2 shows the thermogravimetric curve of the Cu_5%_–MoS_2_ sample, where the weight loss below 100 °C is likely due to moisture evaporation from the material's surface, whereas the weight loss between 240 °C and 400 °C may be attributed to the evaporation of crystallization water. The experimental results demonstrate that Cu_5%_–MoS_2_ is highly stable under the experimental conditions.

**
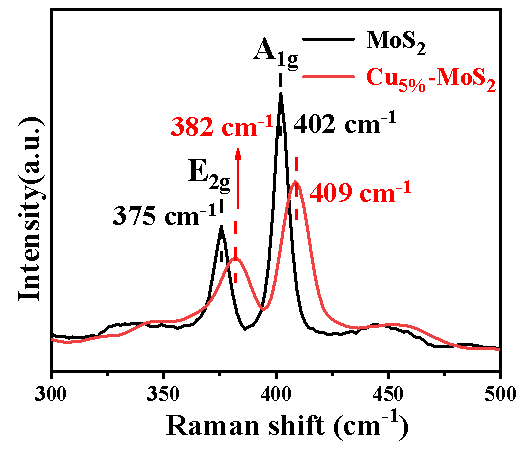
**

**Figure S3.** Raman spectra of Cu_5%_–MoS_2_ and MoS_2_.

The structural characteristics of molybdenum disulfide were analyzed via Raman spectroscopy with a 532 nm laser as the excitation source. In Figure S3, the main characteristic peaks of the MoS_2_ phase appear at 375 cm^–1^ and 402 cm^–1^. After Cu doping, the Raman characteristic peaks slightly redshifted to 382 cm^–1^ and 409 cm^–1^. The significant shift toward higher frequencies and broadening of the full width at half maximum (FWHM) of the Cu-doped MoS_2_ Raman peaks are due to the increased interatomic interactions caused by Cu doping, which altered the coordination environment of the original cations.

**
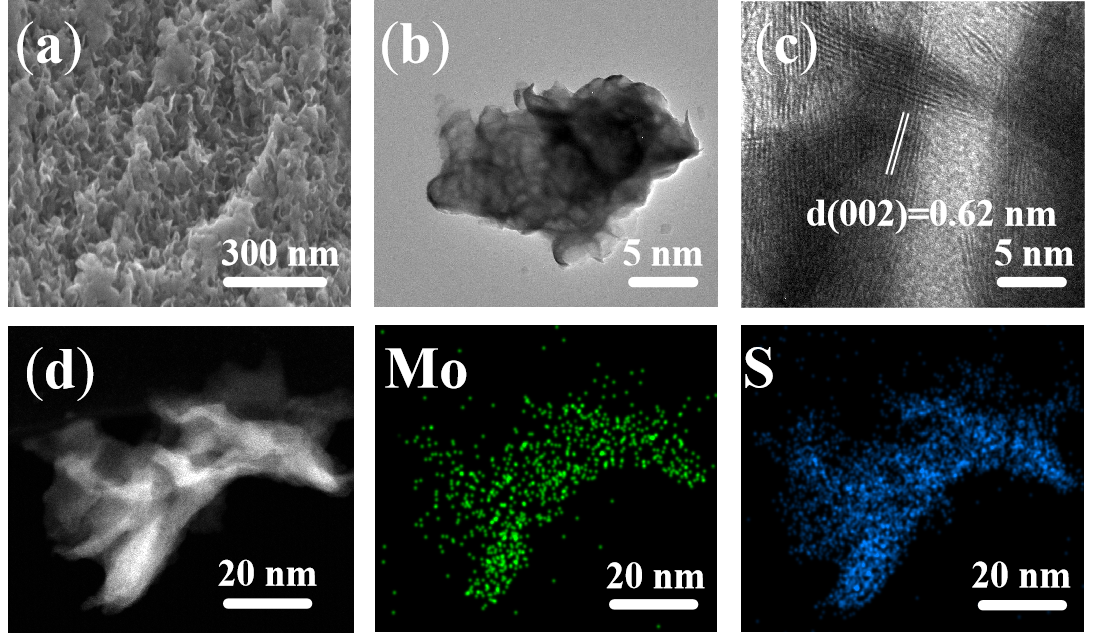
**

**Figure S4.** Morphology of MoS_2_. (a) SEM, (b-c) TEM, (d) element distribution.

As shown in Figure S1, it can be seen from SEM and TEM images that monomer MoS_2_ is nano-flake, lattice spacing d=0.62 nm corresponds to the (002) plane of MoS_2_, and the presence of Mo and S can be observed from EDS.


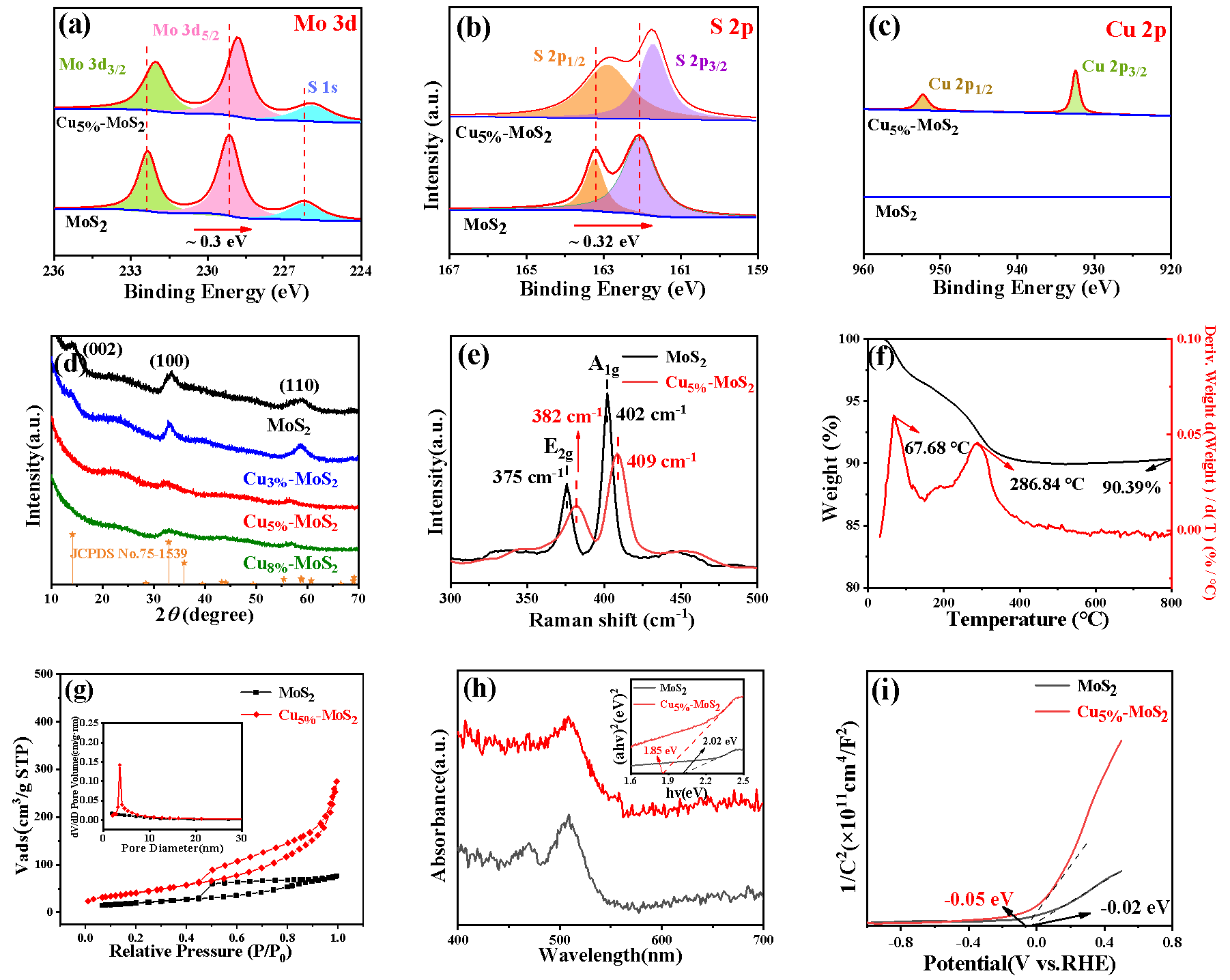


**Figure S5.** (a-c) High-resolution XPS spectra of Mo 3*d*, S 2*p*, and Cu 2*p* of MoS_2_ and Cu_5%_–MoS_2._

_
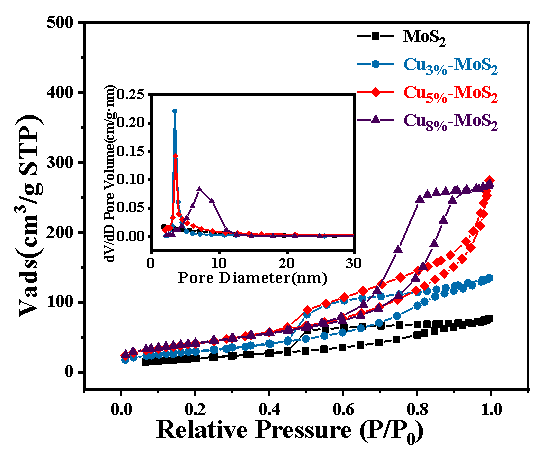
_

**Figure S6.** Nitrogen adsorption isotherms and corresponding pore size distributions of different samples.

N_2_ adsorption analysis (Figure S6) revealed that both MoS_2_ and Cu/MoS_2_ exhibit type І adsorption isotherms and H3-type hysteresis loops [33], which are characteristic of mesoporous materials. The BET surface area of Cu_5%_–MoS_2_ is 154.25 m^2^/g, with a pore size range of 5–25 nm, whereas pure MoS_2_ has a BET surface area of 73.47 m^2^/g. This clearly shows that the incorporation of Cu provides more active sites. These results are consistent with the SEM findings.


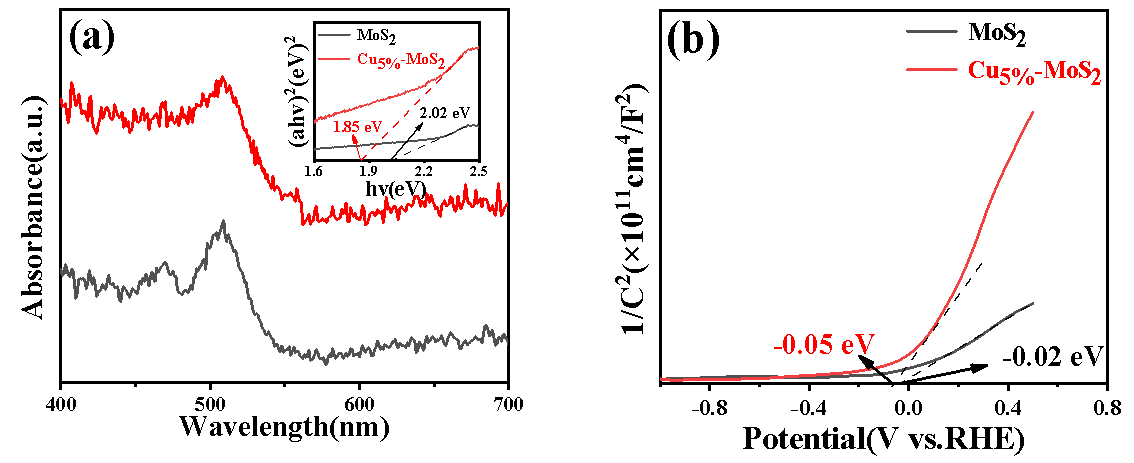


**Figure S7.** (a) UV–vis DRS spectra and corresponding band gap energies (inset) of MoS_2_ and Cu_5%_–MoS_2_, (b) Mott–Schottky (MS) plots of MoS_2_ and Cu_5%_–MoS_2_.

The UV–vis–DRS diffuse reflectance spectra (Figure S7a) and Mott–Schottky (MS) plots (Figure S7b) reveal that the band gaps of MoS₂ and Cu_5%_–MoS_2_ are 2.02 eV and 1.85 eV, respectively, indicating that Cu_5%_–MoS_2_ has a broader light absorption range, which is beneficial for light absorption.

**Table S1.** EXAFS fitting parameters at the Mo K-edge for various samples (*S*_0_^2^=0.89).

| **Sample** | **Shell** | ***CN^a^*** | ***R*(Å)*^b^*** | **σ2 (Å^2^) ^c^** | **Δ*E*_0_(eV)*^d^*** | **R factor** |
| --- | --- | --- | --- | --- | --- | --- |
| **Mo foil** | **Mo-Mo1** | **8** | **2.71** | **0.003±0.001** | **-9.51±0.99** | **0.001** |
|  | **Mo-Mo2** | **6** | **3.12** | **0.003±0.001** |  |  |
| **MoO_2_** | **Mo-O** | **5.28** | **2.01** | **0.002±0.001** | **-3.00±2.28** | **0.015** |
| **MoS_2_** | **Mo-S** | **5.82** | **2.40** | **0.002±0.001** | **-3.72±0.94** | **0.004** |
|  | **Mo-Mo** | **6.30** | **3.17** | **0.002±0.001** |  |  |
| **Cu_5%_-MoS_2_** | **Mo-S** | **4.98** | **2.40** | **0.003±0.001** | **-1.61±0.96** | **0.006** |
|  | **Mo-Mo** | **1.86** | **3.16** | **0.005±0.002** |  |  |

*^a^CN*, coordination number; *^b^R*, distance between absorber and backscatter atoms; *^c^σ*^2^, Debye-Waller factor to account for both thermal and structural disorders; *^d^ΔE*_0_, inner potential correction; *R* factor indicates the goodness of the fit. Fitting range: 3.0 < k (/Å) < 12 and 1 < R (Å)< 3.

_
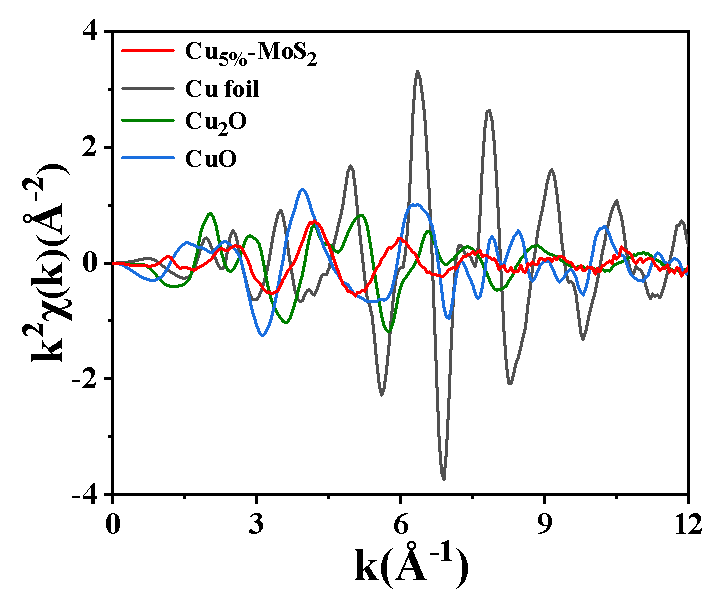
_

**Figure S8.** The Cu EXAFS spectra in k-space of Cu_5%_-MoS_2_.


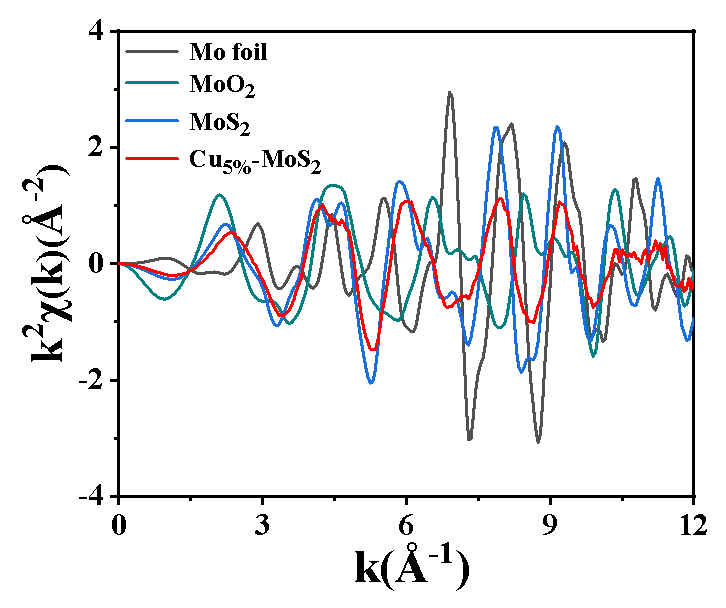


**Figure S9.** The Mo EXAFS spectra in k-space of Cu_5%_-MoS_2_.


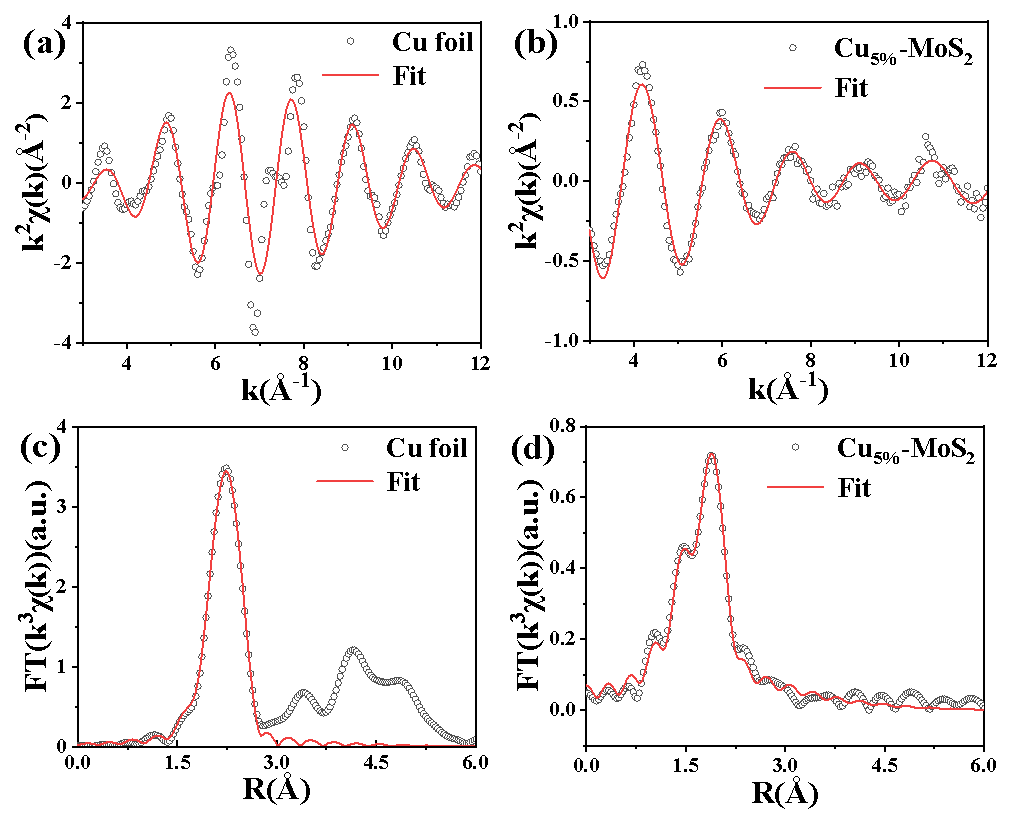


**Figure S10.** The experimental EXAFS function (black circles) and the calculated Cu-Cu and Cu-S contributions (red line) fitting results. (a) The Cu EXAFS spectra in k-space of Cu foil. (b) The Cu EXAFS spectra in k-space of Cu_5%_-MoS_2_


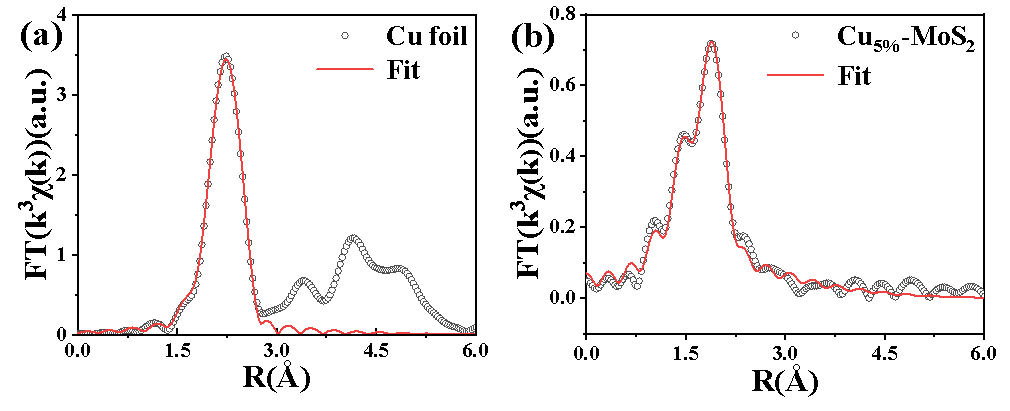


**Figure S11.** The experimental EXAFS function (black circles) and the calculated Cu-Cu and Cu-S contributions (red line) fitting results. (a) The Cu EXAFS spectra in R-space of Cu foil. (b) The Cu EXAFS spectra in R-space of Cu_5%_-MoS_2_.


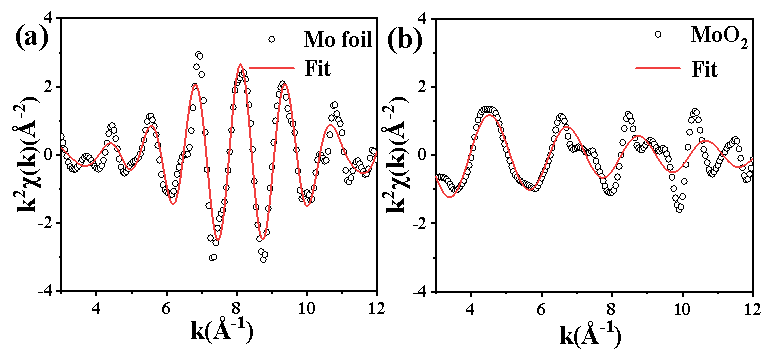


**Figure S12.** The experimental EXAFS function (black circles) and the calculated Mo-Mo and Mo-S contributions (red line) fitting results. (a) The Mo EXAFS spectra in K-space of Mo foil. (b) The Mo EXAFS spectra in K-space of MoO_2_.


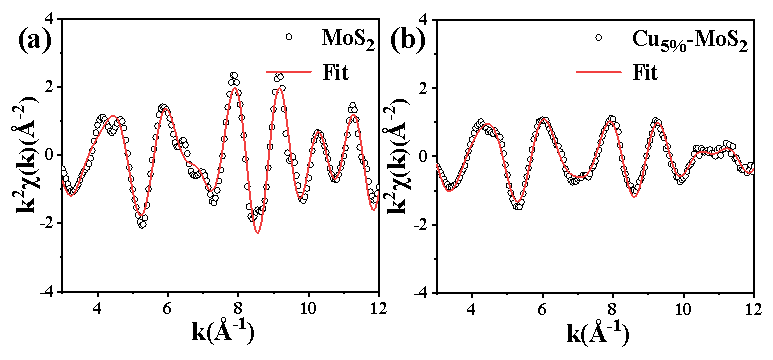


**Figure S13.** The experimental EXAFS function (black circles) and the calculated Mo-Mo and Mo-S contributions (red line) fitting results. (a) The Mo EXAFS spectra in K-space of MoS_2_. (b) The Mo EXAFS spectra in K-space of Cu_5%_-MoS_2_.


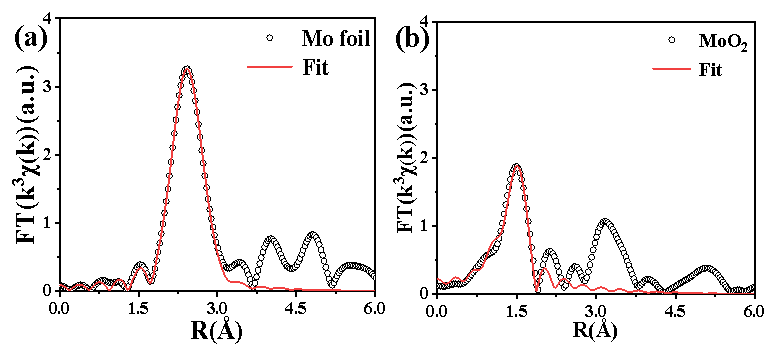


**Figure S14.** The experimental EXAFS function (black circles) and the calculated Mo-Mo and Mo-S contributions (red line) fitting results. (a) The Mo EXAFS spectra in R-space of Mo foil. (b) The Mo EXAFS spectra in R-space of MoO_2_.


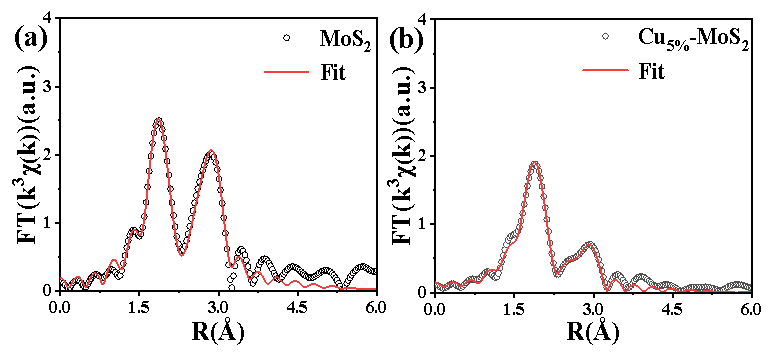


**Figure S15.** The experimental EXAFS function (black circles) and the calculated Mo-Mo and Mo-S contributions (red line) fitting results. (a) The Mo EXAFS spectra in R-space of Cu_5%_-MoS_2_. (b) The Mo EXAFS spectra in R-space of Cu_5%_-MoS_2_.


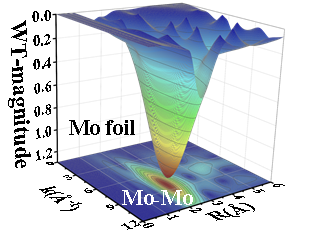


**Figure S16.** Full-range EXAFS WT plot of Mo foil.


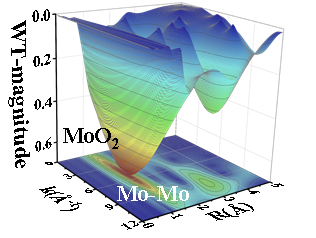


**Figure S17.** Full-range EXAFS WT plot of MoO_2_.


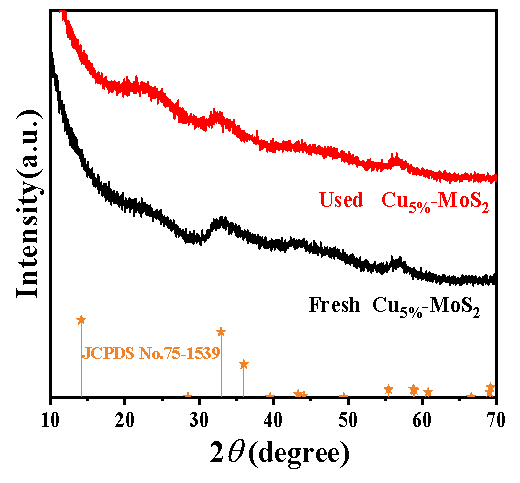


**Figure S18.** XRD patterns of Cu_5%_–MoS_2_ before and after the reaction.

The stability of Cu_5%_–MoS_2_ before and after the reaction is proved by XRD test. The experimental results show that the peak position and strength of the XRD pattern of the catalyst before and after the reaction do not change significantly, that is, the catalyst has a certain stability.


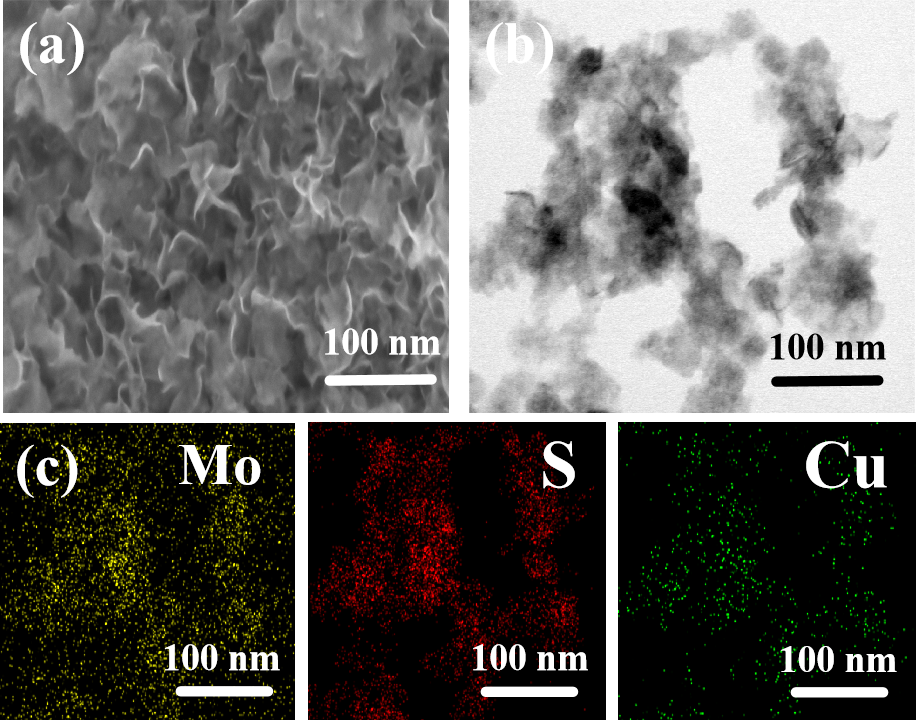


**Figure S19.** (a) SEM images; (b) TEM images and (c) elemental distribution of Cu_5%_–MoS_2_ after the reaction.

SEM, TEM and EDS tests were carried out on the lower Cu_5%_–MoS_2_ after the reaction. The experimental results show that the Cu_5%-_MoS_2_ after the reaction is still nano-flake, which has good stability.


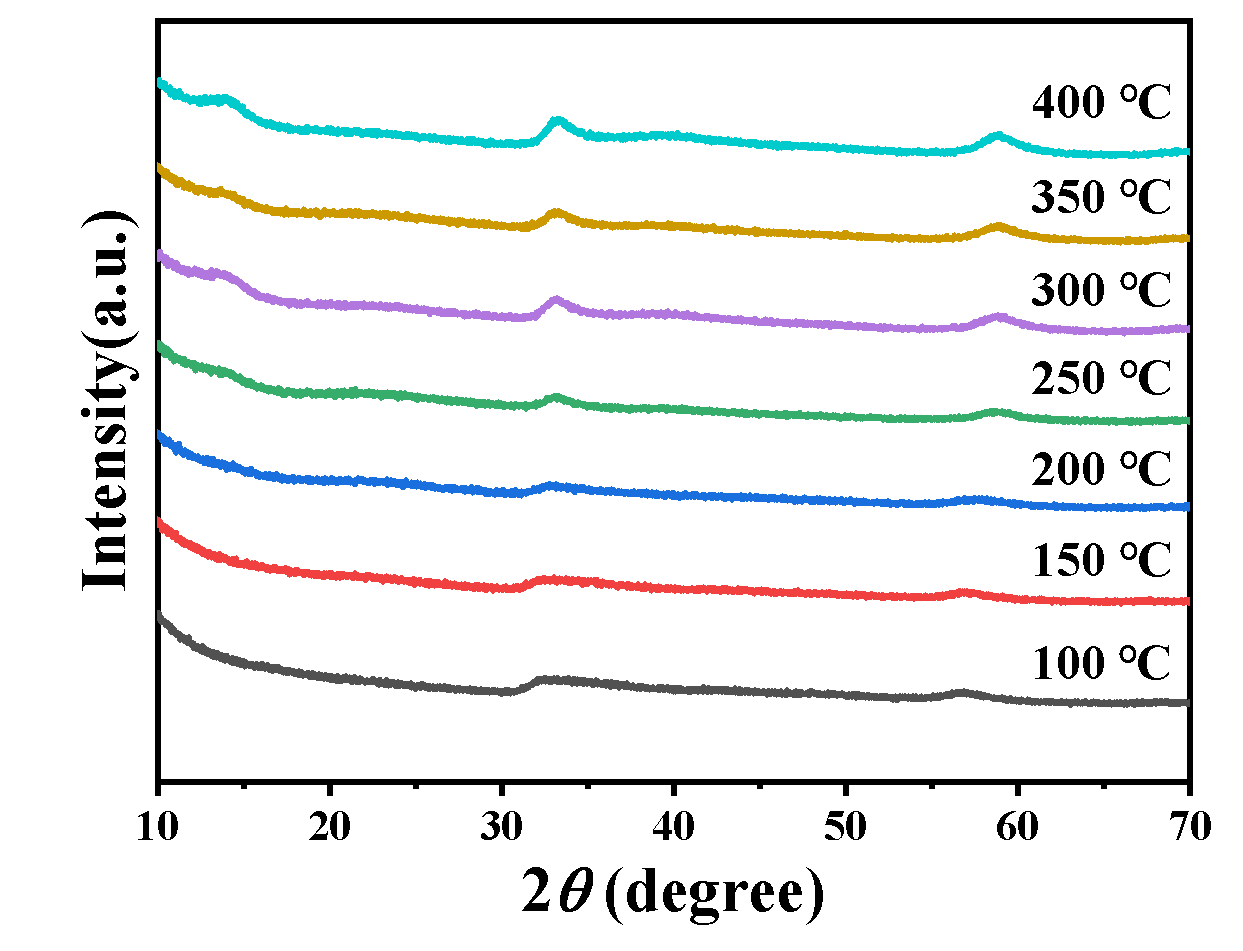


**Figure S20.** XRD patterns of MoS_2_ calcined at different temperatures in Ar atmosphere.

In order to study the performance of pure MoS_2_, MoS_2_ was calcined under Ar atmosphere for 2 h at different temperatures for XRD test. XRD results show that different temperature calcination has no significant effect on the crystal form of MoS_2_.


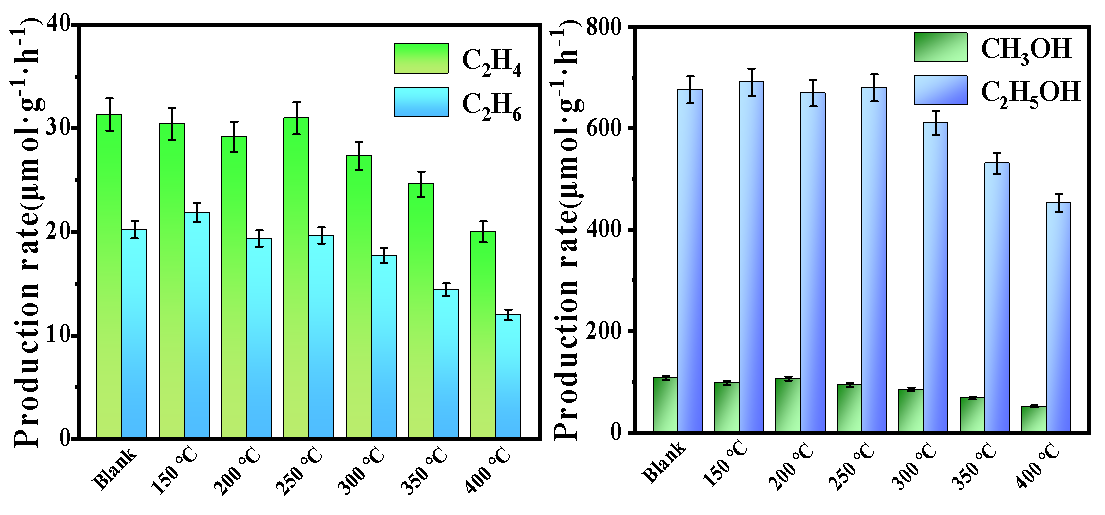
**Figure S21.** Activity test of MoS_2_ calcined at different temperatures in an Ar atmosphere.

In order to study the pure performance, the activity test was carried out after calcination at different temperatures for 2 h under Ar atmosphere. The experimental results showed that calcination at different temperatures had a certain impact on the catalytic performance of the catalyst. The decrease of catalytic performance at 400 ℃ may be due to the volatilization of the material crystal water, which was consistent with the results of thermogravimetric experiments.

**Table S2.** The light intensity corresponding to different wavelengths

| **Wavelength/nm** | **Optical power intensity/mW** | **Optical power density/(mW/cm^2^)** |
| --- | --- | --- |
| **320-780** | **715** | **903** |
| **365** | **90** | **116** |
| **400** | **84** | **106** |
| **450** | **108** | **136** |
| **500** | **96** | **120** |
| **550** | **88** | **112** |

It has been discovered that the light source exhibits peak optical power intensity and optical power density within the 320-780 nm range, aligning with the experimental findings. Conversely, the optical power intensity and optical power density of bandpass plates decrease significantly across various wavelengths, owing to their ability to transmit only light of specific, fixed wavelengths.


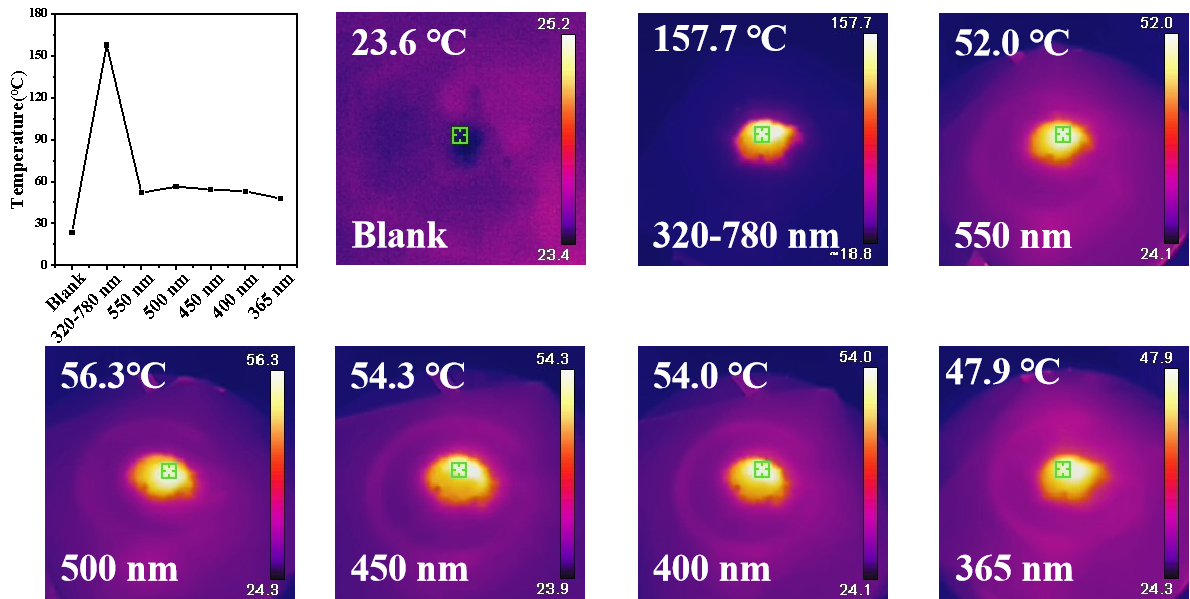


**Figure S22.** Under unheated conditions, the thermal image of Cu_5%_–MoS_2_ illuminated by light sources of different wavelengths stabilizes within 5 minutes.


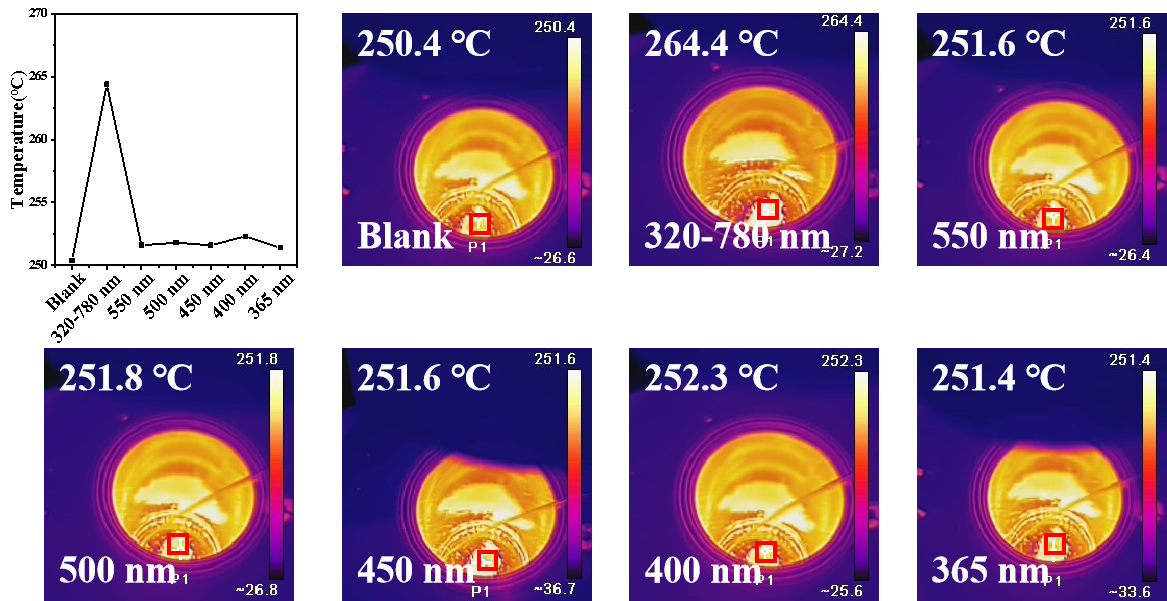


**Figure S23.** Under heated conditions, the thermal image of Cu_5%_–MoS_2_ illuminated by light sources of different wavelengths stabilizes within 5 minutes.


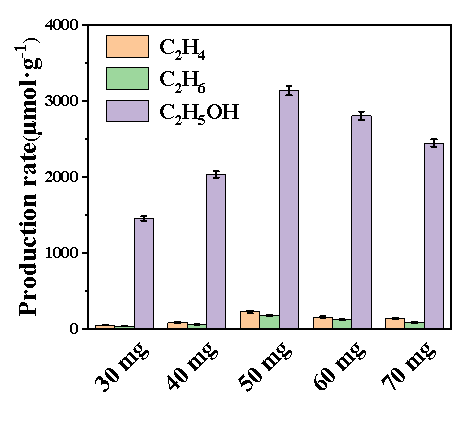


**Figure S24.** The influence of different masses of Cu_5%_-MoS_2_ on catalytic activity.

In addition, during the catalytic process, the amount of catalyst used and the duration of the catalytic reaction have a significant impact on the final catalytic effect. We conducted catalytic experiments with different loadings of the Cu_5%_–MoS_2_ catalyst (Figure S24), and found that the catalytic effect increased with the increase in catalyst loading, with 50 mg exhibiting the best catalytic performance. However, when the catalyst loading was further increased, the yield of C_2+_ did not continue to increase and even decreased slightly. Excessive catalyst may hinder the mass and heat transfer within the reaction system, reducing the contact efficiency between the reactants and the catalyst. When the amount of catalyst is reduced, the number of catalyst active centers per unit volume or per unit mass is correspondingly reduced, which leads to a lower reaction rate. A reduction in the reaction rate means that less of the reactant is converted to the product in the same amount of time, so the yield per gram of catalyst is also reduced.


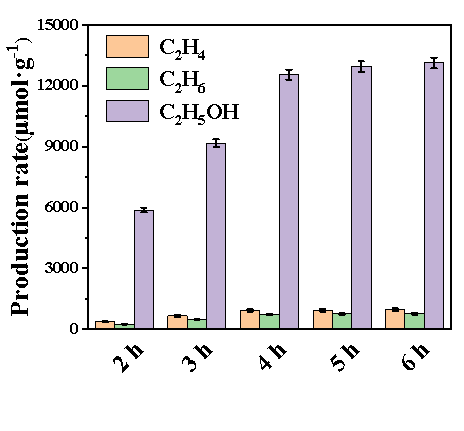


**Figure S25.** The influence of different reaction times of Cu_5%_-MoS_2_ on catalytic activity.

Additionally, experiments were conducted to investigate the effect of reaction time on activity, and the results are shown in Figure S25. The yield of C_2_ products gradually increased with increasing reaction time, with 4 h identified as the optimal duration; further increases in the reaction time did not significantly increase the yield. This phenomenon is attributed to the continuous consumption of carbon dioxide in the reaction system with the increase of reaction time, and when the concentration is too low, it is not enough to continue to support the further occurrence of the reaction, and the reaction equilibrium is reached. So there was no significant increase in the product after 4 h.


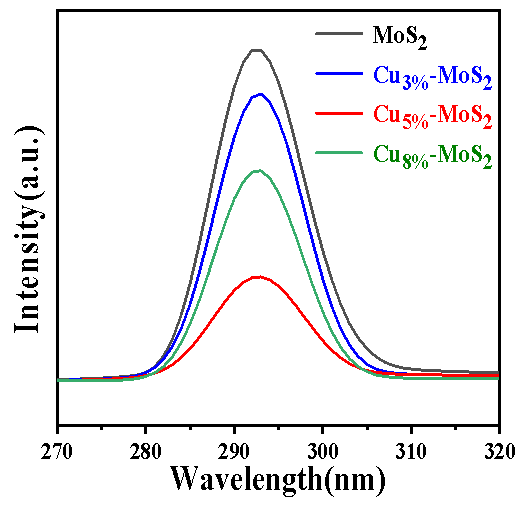


**Figure S26.** Fluorescence spectra of Cu/MoS_2_ with varying Cu loadings.

Figure S26 shows the PL spectra of the samples. Compared with that of pure MoS_2_, the peak intensity of Cu/MoS_2_ is lower, indicating a higher separation efficiency of photogenerated carriers in Cu/MoS_2_. The introduction of new intermediate energy levels by Cu atoms within the band structure of the catalyst is the underlying cause. These levels, situated typically between the conduction band and the valence band, effectively capture photogenerated electrons or holes, subsequently prolonging their lifespans and minimizing the likelihood of recombination.


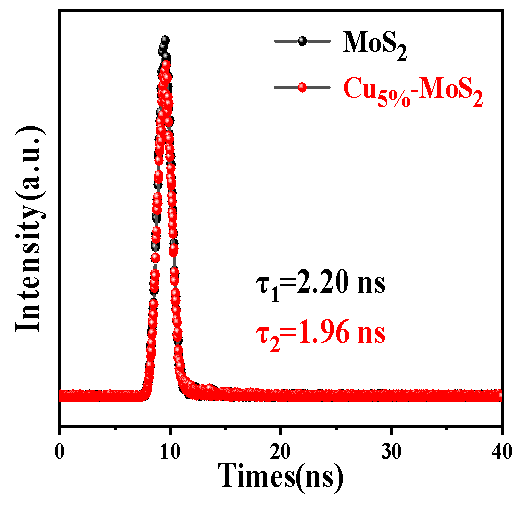


**Figure S27.** Transient fluorescence spectra of MoS_2_ and Cu_5%_-MoS_2_.

Transient fluorescence was used to investigate the fluorescence lifetime of the catalysts. After Cu doping, the fluorescence lifetime of the catalyst decreased from 2.20 ns to 1.96 ns (Figure S26). Carrier migration is associated with fluorescence quenching, leading to a reduced fluorescence lifetime. The experimental results suggest that the addition of Cu facilitates more efficient carrier transfer. The results were consistent with those of photoluminescence (PL) spectroscopy.


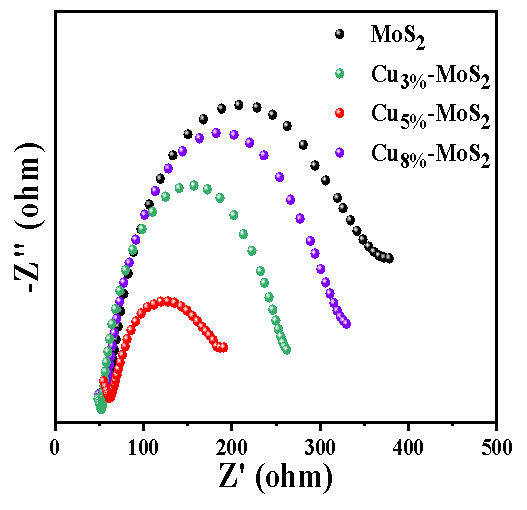


**Figure S28.** Electrochemical impedance spectra of MoS_2_ and Cu/MoS_2_.

As shown in Figure S28, the catalyst doped with Cu has a smaller semicircle radius than does pure MoS_2_, indicating a lower resistance. The decrease in impedance and increase in the separation of electrons and holes in MoS_2_ can be attributed to the role of Cu as an electron donor. Specifically, Cu, with its unique valence electron structure, introduces additional free electrons into MoS_2_ during the doping process, thereby increasing the carrier concentration. This, in turn, leads to a significant decrease in impedance and a more efficient separation of electrons and holes.


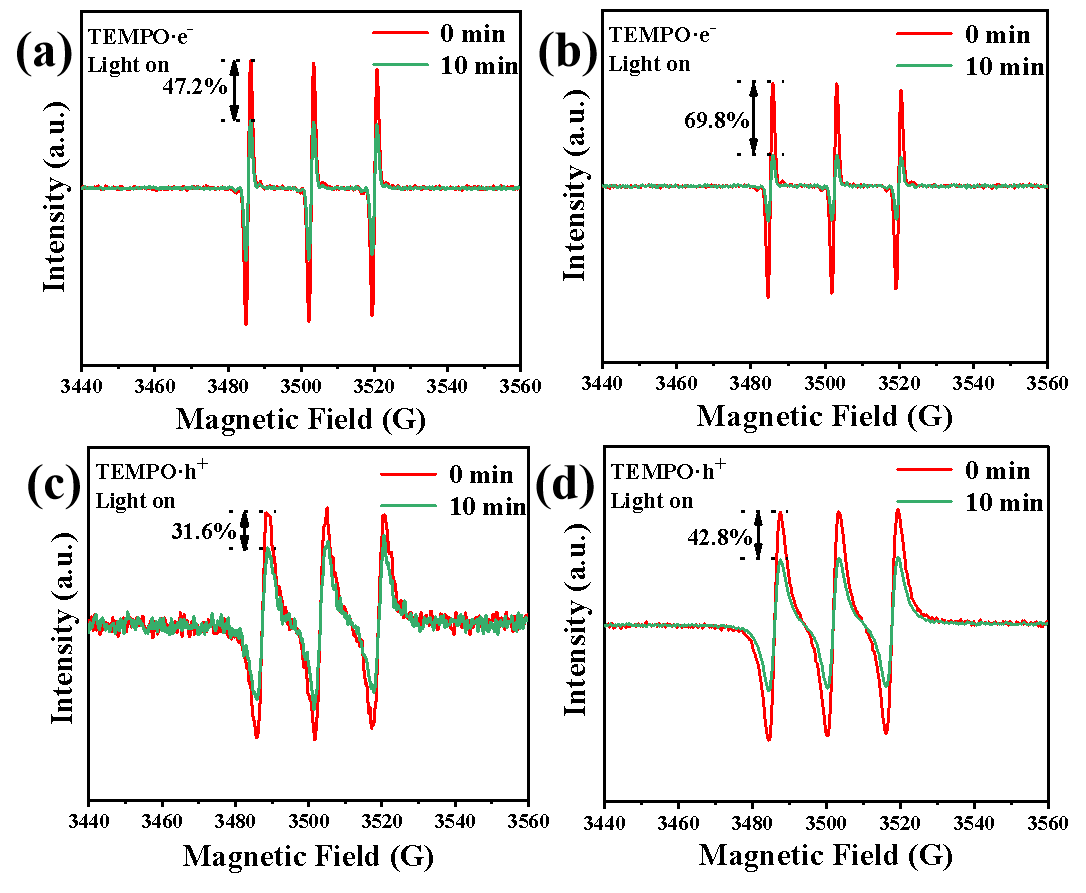


**Figure S29.** (a–d) EPR experiments with TEMPO as an h⁺ and e⁻ scavenger for MoS_2_ and Cu_5%_–MoS_2_.

We measured the EPR spectra of MoS_2_ and Cu_5%_–MoS_2_ to study the ability of the catalysts to capture electrons (e⁻) and holes (h⁺). TEMPO, a paramagnetic substance, exhibits a 1:1:1 triplet peak in EPR tests. When photogenerated e⁻ and h⁺ are produced on the catalyst surface, they react with TEMPO, causing a decrease in the TEMPO signal. A 1 μmol/L aqueous solution of TEMPO was used as an electron scavenger. After 10 min of light exposure to MoS_2_ in the TEMPO solution, the EPR signal peak decreased by 47.2% (Figure S29a), indicating an efficient reaction between the photogenerated e⁻ of MoS_2_ and TEMPO, weakening the TEMPO signal. For the Cu_5%_–MoS_2_ sample, the signal peak reduction was even greater (69.8%) under the same conditions (Figure S29b). Additionally, a 1 μmol/L acetonitrile solution of TEMPO was used as a hole scavenger. After 10 min of light exposure, the TEMPO signal for MoS_2_ decreased by 31.6% (Figure S29c), whereas the signal for Cu_5%_–MoS_2_ decreased by 42.8% (Figure S29d), suggesting that more h⁺ reacted with TEMPO in the Cu_5%_–MoS_2_ sample. These results confirm that Cu_5%_–MoS_2_ generates more photogenerated e⁻ and h⁺ under light, enhancing the photocatalytic performance.


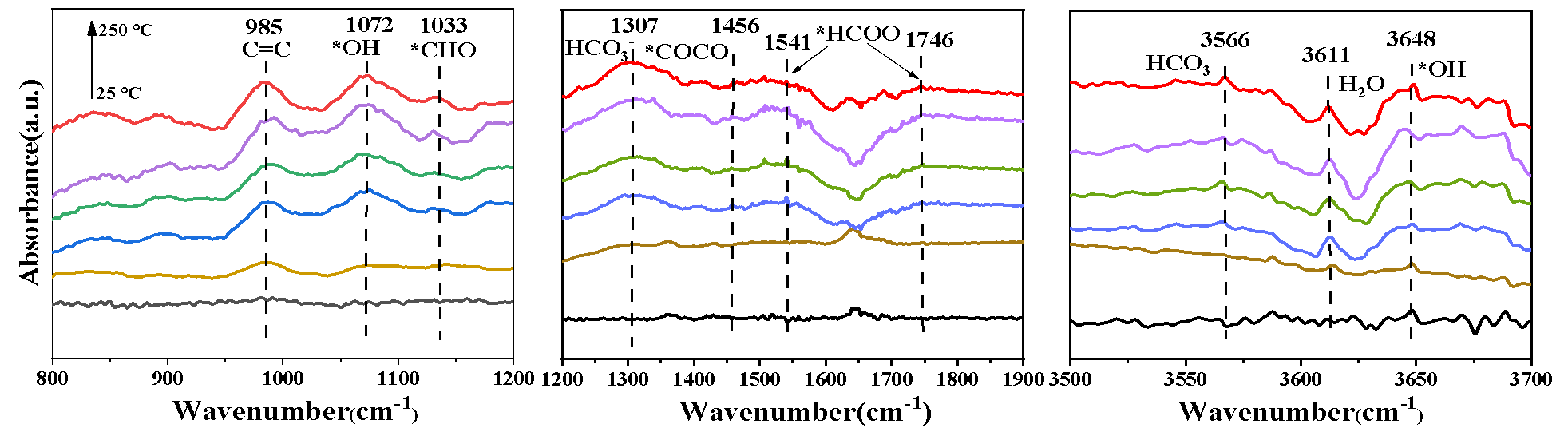


**Figure S30.** (a) In situ FTIR spectroscopy characterization of coadsorption of CO_2_ and H_2_O vapor mixtures on MoS_2_ under light irradiation.

To gain a deeper understanding of the possible reaction mechanisms, in-situ FTIR spectroscopy analysis was conducted during the photothermal reduction of CO_2_ over MoS_2_. As shown in Figure S30, an infrared peak at 1307 cm⁻¹ was detected, which is attributed to the *COCO group, a crucial primary intermediate in the reduction of CO_2_. Simultaneously, peaks corresponding to H_2_O (3611 cm⁻¹) and HCO_3_⁻ (3556 cm⁻¹) were observed, confirming the adsorption of H_2_O and CO_2_ on the catalyst. Additionally, the presence of C=C (985 cm⁻¹) indicates the formation of C_2+_ products. At 1033 cm⁻¹, adsorbed *CHO was detected. The absorption peak near 1072 cm⁻¹ belongs to the *OH group, while the peak at 1456 cm⁻¹ can be inferred as the asymmetric stretch of the *COCO group. Furthermore, the peaks at 1541 cm⁻¹ and 1746 cm⁻¹ can be attributed to the characteristic peaks of the *CH_3_CHO group.


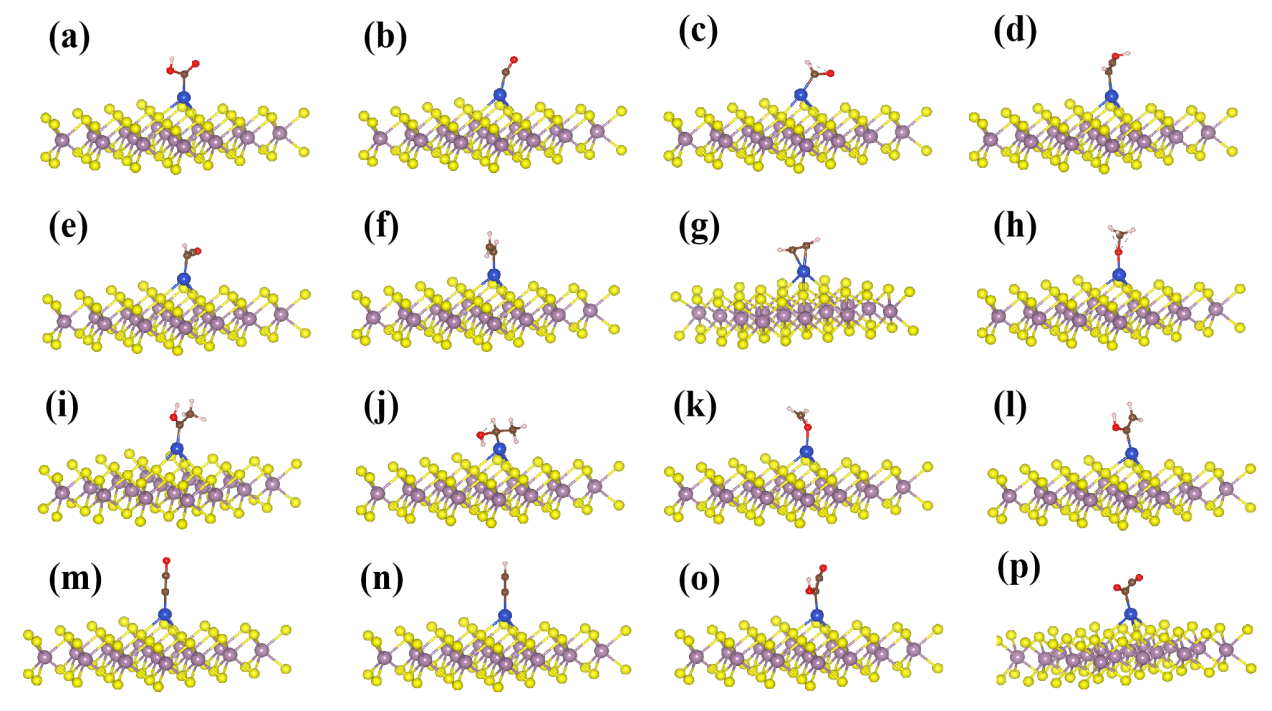


**Figure S31.** Intermediate configurations for CO_2_ adsorption and reduction on (a-p) Cu_5%_-MoS_2_ surfaces. Purple, yellow, red and black spheres represent the Mo, S, O and C atoms, respectively.


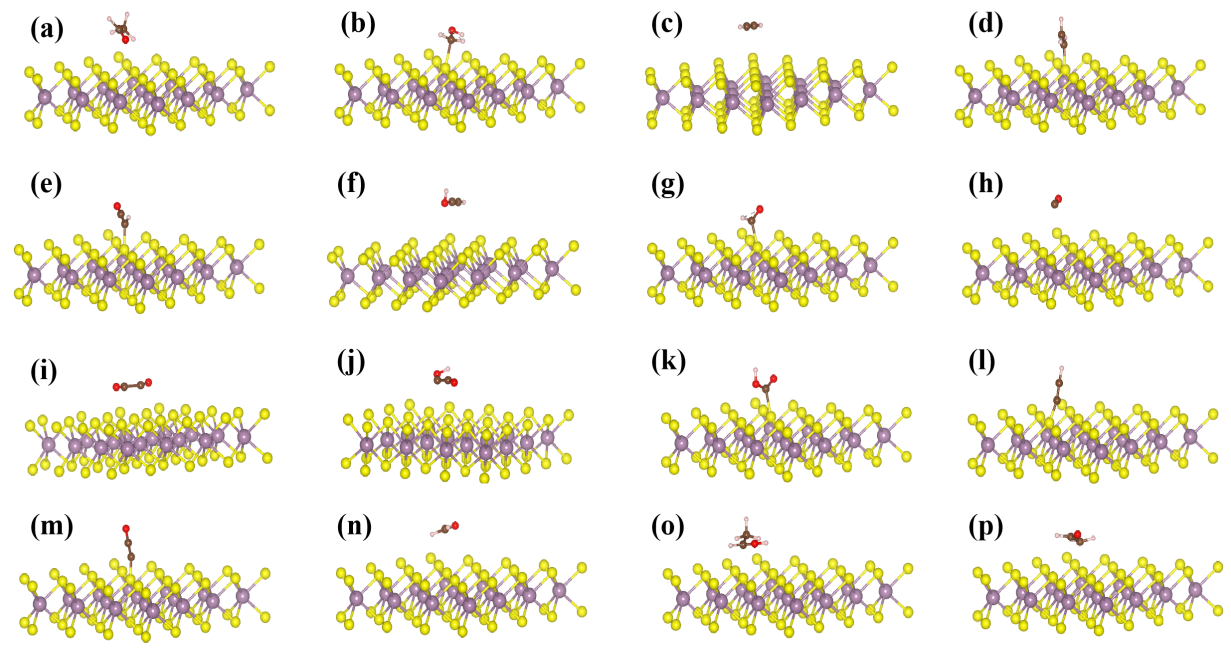


**Figure S32.** Intermediate configurations for CO_2_ adsorption and reduction on (a-p) MoS_2_ surfaces. Purple, yellow, blue, red and black spheres represent the Mo, S, Cu, O and C atoms, respectively.

**Table S3.** The light intensity corresponding to different wavelengths

| **Different samples** | **Actual copper content** |
| --- | --- |
| **Cu_3%_-MoS_2_** | **3.347%** |
| **Cu_4%_-MoS_2_** | **3.627%** |
| **Cu_5%_-MoS_2_** | **4.6616%** |
| **Cu_6%_-MoS_2_** | **5.846%** |
| **Cu_8%_-MoS_2_** | **8.415%** |

The specific content of Cu/MoS_2_ in different proportions is shown in Table S3 of supplementary materials, and the test method is as follows:

ICP-OES:

Model :AGILENT ICP-OES 730

Conditions:

1. Qualitative analysis: Full spectrum scanning of unknown samples is carried out to give the types of inorganic elements (including non-metallic phosphorus and sulfur elements) and the range of element content in the samples. 2. Quantitative analysis Accurate quantitative analysis of the element content in the sample, the detection element lower limit is 0.1 mg/kg, the upper limit is 20%. Elements beyond the scope should be analyzed in conjunction with other analytical methods

Conventional digestion - Microwave digestion:

A certain amount of samples were weighed into a PTFE container, added with 5 mL concentrated nitric acid, 3 mL HC1, 1 mL HF, 2mLH_2_O_2_, and sealed in a microwave digestion furnace. The samples were heated at 1200 W for 20 min to 130 ℃ and kept for 5 min, and heated for 20 min to 180 ℃ and kept for 40 min. Cool to room temperature

Test:

The cooled solution was transferred to a 25 mL plastic volumetric bottle, and finally filled with deionized water. The dissolved solution was tested successively, and the diluted solution beyond the curve was tested again.

Standard test solution: the standard solution is the national standard material, and the curve concentration points are 0, 0.5, 1.0, 2.0, 5.0 mg/L, respectively.
